# Myopia & painful muscle form of temporomandibular disorders: connections between vision, masticatory and cervical muscles activity and sensitivity and sleep quality

**DOI:** 10.1038/s41598-023-47550-6

**Published:** 2023-11-19

**Authors:** Grzegorz Zieliński, Anna Matysik-Woźniak, Michał Baszczowski, Maria Rapa, Michał Ginszt, Beata Pająk, Jacek Szkutnik, Robert Rejdak, Piotr Gawda

**Affiliations:** 1 Department of Sports Medicine, Medical University of Lublin, Lublin, Poland; 2 Department of General and Pediatric Ophthalmology, Medical University of Lublin, Lublin, Poland; 3 Interdisciplinary Scientific Group of Sports Medicine, Department of Sports Medicine, Medical University of Lublin, Lublin, Poland; 4 Students’ Scientific Association at the Department and Clinic of General and Pediatric Ophthalmology, Medical University of Lublin, Lublin, Poland; 5 Department of Rehabilitation and Physiotherapy, Medical University of Lublin, Lublin, Poland; 6 Department of Functional Masticatory Disorders, Medical University of Lublin, Lublin, Poland

**Keywords:** Anatomy, Health care, Risk factors, Diseases, Dental diseases, Eye diseases, Oral diseases

## Abstract

The main aim of this study is to evaluate the effects of painful muscle form of temporomandibular disorders and myopia on the connections between the visual organ, the bioelectrical activity and sensitivity of the masticatory and cervical muscles, and sleep quality. Subjects were divided into 4 groups (Myopia & TMDs, Myopia (Without TMDs), Emmetropic & TMDs and Emmetropic (Without TMDs)). The study was conducted in the following order of assessment: examination for temporomandibular disorders, assessment of the muscle activity by electromyograph, pressure pain thresholds examination, ophthalmic examination and completion of the Pittsburgh Sleep Quality Index. It was observed that the Myopia & TMDs group had higher muscle tenderness, higher resting and lower functional muscle bioelectrical activity. The visual organ is clinical related to the masticatory and cervical muscles. TMDs and myopia alter masticatory and cervical muscle activity. The thickness of the choroid in people with myopia is related to muscle tenderness. TMDs and myopia impair sleep quality. It is recommended to determine the number of people with refractive error and its magnitude in the sEMG study in order to be able to replicate the research methodology.

## Introduction

Myopia is caused by the abnormal focusing of light rays in front of the retina^1^. It is suggested that there will be 4758 million people with myopia in 2050^2^. Epidemiological data suggest a prevalence of low myopia up to 31.62% and high myopia up to 20.12% in adolescents^3^. Myopia is one of the most prevalent refractive errors^4^. The high prevalence rates pose a serious public health challenge due to refractive error^5^.

Another serious social problem is temporomandibular disorders (TMDs)^6^. TMDs include issues related to the masticatory muscles, temporomandibular joints and surrounding tissues^7^. TMDs are the most common type of non-dentogenic craniofacial pain and can predispose to chronic pain^8^. An estimated 11.2–12.4 million US adults (4.8 percent of the population) had temporomandibular joint pain in 2018^9^.

Studies suggest links between the musculo-fascial system and the organ of vision^10, 11^. According to a recent study (2022), positive correlations were observed between bioelectrical muscle activity (of the digastric muscle) and the length of the eyeball on the same side^12^. In addition, another study (2022) using surface electromyography (sEMG) observed that bioelectrical activity within the anterior temporalis muscle appears to be related to axial length of eyeball (negative correlations), retinal thickness (negative correlations) and choroidal thickness (positive correlations) in women with myopia^13^. It has also been observed that there is an immediate response between eye closure and a change in bioelectrical activity on the masticatory muscles in myopic subjects^14, 15^. Such a response was not observed in emmetropic subjects^16^.

Studies have recognized links between refractive error and sleep quality^17, 18^ and also between TMDs and sleep quality^19, 20^. Sleep problems are a common and significant social health concern^21^. It is estimated that nearly one-third of the general population experiences symptoms of insomnia (defined as difficulty falling asleep and/or maintaining sleep)^22^. There is an association between obstructive sleep apnea and TMDs^23, 24^. Inadequate and/or disturbed sleep in patients with obstructive sleep apnea can increase pain sensitivity, also obstructive sleep apnea contributes to hypoxemia, which increases inflammatory cytokines, contributing to the pathogenesis of many comorbidities^23, 24^. This can directly affect the development of TMDs, muscle changes and pain sensitivity.

The etiology of myopia^25^, TMDs^26^ and sleep problems is multifactorial^27^. There are hypotheses of a muscular component in the etiology of myopia^13^, also TMDs has a muscle form^28^, and it has been proven that good sleep quality is associated with greater muscle strength^29^.

Given the above information, the public health significance of myopia and TMDs and the impact of the muscular component, the authors decided to conduct the present study. The main aim of this study is to evaluate the effects of painful muscle form of TMDs and myopia on the connections between the visual organ, the bioelectrical activity and sensitivity of the masticatory and cervical muscles, and sleep quality. It was hypothesized that the painful muscle form of TMDs and myopia would influence on the bioelectrical activity and sensitivity of the masticatory and cervical muscles, and also sleep quality. An additional aim was to develop additional guidelines for electromyographic examination of patients with ocular problems. To the best of the authors knowledge, this is the first study of its kind.

## Methods and materials

Two hundred and one people were invited to participate in the study. Written informed consent was obtained from all participants who took part in the study. Participants knew the objectives of the study and could withdraw from the study at any time. The study was conducted at the Department of General and Pediatric Ophthalmology and the Department of Functional Masticatory Disorders at the Medical University of Lublin.

Inclusion criteria used in the groups were:

- The first group—Myopia & TMDs:
  - Myopia (defined as a refractive error ≤ − 0.50 diopters (D)^30^),
  - Best corrected visual acuity of 1.0,
  - Painful muscle form of TMDs,
  - Full dentition.
- The second group—Myopia (Without TMDs):
  - Myopia (defined as a refractive error ≤ − 0.50 diopters (D)^30^),
  - Best corrected visual acuity of 1.0,
  - No TMDs,
  - Full dentition.
- The third group—Emmetropic & TMDs:
  - Emmetropia,
  - Visual acuity of 1.0,
  - Painful muscle form of TMDs,
  - Full dentition.
- The fourth group—Emmetropic (Without TMDs):
  - Emmetropia,
  - Visual acuity of 1.0,
  - No TMDs,
  - Full dentition.

The following exclusion criteria were used in the clinical examination: hyperopia, ocular diseases, optic nerve diseases, intraocular pressure greater than 20 mmHg, eye surgery, any type of malocclusion (clinical examination according to the British Standard Institute classification of malocclusion^31–33^ and the measurement of overbite and overjet was performed with a caliper), class II and III according to Angle’s classification, active or in the last 6 months completed orthodontic treatment, muscle hypertrophy of the tested muscles, trauma and surgical treatment in the head and neck region within the last 6 months before the examination, any inflammation within the oral cavity, taking medications in the last two weeks before the study (including muscle relaxants, steroids, painkillers, anti-inflammatories), neurological disorders in the head and neck region, neoplastic diseases (regardless of type and location), pregnancy, presence of active trigger points (the following diagnostic criteria according to Travell & Simons) in the examined muscles^34^.

The study was conducted in the following order of assessment: examination for temporomandibular disorders, assessment of the muscle activity, pressure pain thresholds examination, ophthalmic examination and finally completion of the Pittsburgh Sleep Quality Index (PSQI). Based on the exclusion criteria and the 201 performed tests, 78 subjects—a total of 156 eyeballs—were included in the analysis. Subjects were divided into 4 groups (Myopia & TMDs, Myopia (Without TMDs), Emmetropic & TMDs and Emmetropic (Without TMDs) (Table 1). Personal data such as age, gender, anthropometric data (height and weight) were collected using a self-report questionnaire.

**Table 1:** Results of sEMG repeatability tests.

		10 participants (5 women and 5 men)			The same 10 participants (5 women and 5 men)				
		Mean	SD		Mean	SD	test	*p*	ES
Resting mandibular position	TA	*2.35*	*1.05*	5-min rest between test activities	*2.32*	*1.07*	T	0.07	*0.94*
	MM	*2.69*	*1.34*		*2.63*	*1.35*	T	0.09	*0.93*
	SCM	*3.19*	*2.19*		*3.12*	*2.18*	T	0.07	*0.94*
	DA	*2.63*	*1.15*		*2.56*	*1.14*	T	0.15	*0.89*
The maximum voluntary clenching in intercuspal position	TA	*1.47*	*0.41*		*1.46*	*0.42*	T	0.04	*0.97*
	MM	*1.48*	*0.50*		*1.47*	*0.53*	T	0.01	*0.99*
	SCM	*1.75*	*0.58*		*1.69*	*0.50*	Z	0.57	*0.57*
	DA	*1.76*	*0.53*		*1.70*	*0.52*	T	0.25	*0.81*
The maximum voluntary clenching on dental cotton rolls in intercuspal position	TA	*131.11*	*61.53*		*134.23*	*61.00*	Z	− 0.26	*0.79*
	MM	*135.09*	*55.92*		*139.23*	*55.20*	T	− 0.17	*0.87*
	SCM	*151.03*	*74.99*		*155.11*	*74.89*	T	− 0.12	*0.90*
	DA	*166.18*	*92.55*		*174.29*	*96.86*	T	− 0.19	*0.85*
The pain free maximum unassisted opening	TA	*17.77*	*12.85*		*18.33*	*13.64*	T	− 0.09	*0.93*
	MM	*16.49*	*11.10*		*17.67*	*12.96*	T	− 0.22	*0.83*
	SCM	*26.79*	*16.31*		*27.31*	*16.57*	T	− 0.07	*0.94*
	DA	*26.18*	*22.86*		*27.03*	*24.03*	Z	− 0.26	*0.79*

SD, standard deviation; TA, the temporalis muscle; MM,the masseter muscle; SCM, the sternocleidomastoid muscle; DA, the digastric muscle; T, the Student’s t-test; Z, Mann–Whitney U test.

Significant values are italic.

### Examination of temporomandibular disorders

During patient classification, the measurement of overbite and overjet was performed by a dentist directly with a caliper in all subjects as part of a clinical, functional assessment according to the Polish version of the RDC/TMD examination^35–37^. Overbite and overjet values between 2 and 3 mm were considered normal^38–40^. The examination for the painful muscle form of temporomandibular disorders (TMDs) was conducted by a dentist with a specialization in dental prosthetics (seventh author). The Research Diagnostic Criteria for Temporomandibular Disorders RDC/TMD^7^ was used for the analysis. The RDC/TMD is comprised of three parts: the first is administering part, next is the clinical examination specifications and the last part is the algorithms for the evaluation. The first part is the personal questionnaire and the clinical examination form, the second part describes the clinical examination instructions and verbal guidelines for the patient^7, 41^. In terms of clinical examination: measurement of the range of motion of mandible, evaluation of acoustic symptoms accompanying mandibular movements and palpation of the masticatory muscles and temporomandibular joints^7, 41^.

### Assessment of the muscle activity

An 8-channel BioEMG III electromyograph compatible with the BioPAK measurement system (BioResearch Associates, Inc., Milwaukee, WI, USA) was used to assess muscle bioelectrical activity. The study was conducted between 8 and 12 a.m. to avoid diurnal variation in bioelectrical activity. The electromyographic study was conducted with the subjects’ eyes open, due to the replication of muscle activity during the day, rather than sleep^15, 42^. A standard procedure of skin cleansing with 90% ethanol was used. Disposable Ag/AgCl electrodes with a conductive area of 16 mm were placed on the skin sequentially. The electrodes were placed according to the standards of the SENIAM program (surface EMG for non-invasive assessment of muscles)^43^. The subjects assumed positions with their cervical region supported in a dental chair^12, 13^. The reference electrode (Ag/AgCl conductive surface of 16 mm and diameter of 30 mm) was placed on the forehead^15, 33, 44^. The maximum accepted impedance of the electrodes was determined to < 10^4^ Ohms^45^.

Four pairs of muscles were analyzed:

- The anterior part of the temporalis muscle (TA),
- The superficial part of the masseter muscle (MM),
- The anterior belly of the digastric muscle (DA),
- The middle part of the sternocleidomastoid muscle (SCM)^14, 15, 46^.

The sEMG activity was recorded during:

- Resting mandibular position (10 s),
- The maximum voluntary clenching in intercuspal position (3 clenches of 3 s each with a 2 s break),
- The maximum voluntary clenching on dental cotton rolls in intercuspal position (3 clenches of 3 s each with a 2 s break),
- The pain free maximum unassisted opening (3 abductions of 3 s with a 2 s rest between) ^14, 15^.

The sEMG recording was visually assessed consecutively BioPAK noise tests were performed. The BioEMG III electromyograph is embedded with optically isolated differential amplifiers with the 10^11^ Ohms input impedance of the amplifiers^47^. According to standard signal processing, the signal were amplified with a minimum noise up to 5000 times stronger than their original level^14, 48^. The noise was attenuated to 170 dB using a NoiseBuster digital filter in the BioPAK software. This is a filter that automatically removes 99% of the remaining noise at frequencies higher than 50/60 Hz passed recorded during the examination^47, 48^. According to the methodology of Rój et al. common mode rejection ratio from 120 to 130 dB at 60 Hz^48^. The input common-mode voltage range from − 3.0 to + 3.0 V DC, and a band width between 30 and 1000 Hz^47, 49^. Repeatability was carried out according to a previously published study^46^. Reproducibility of the sEMG protocol was tested by duplicate sEMG measurements on 10 participants (5 women and 5 men). Two independent sEMG measurements were separated by a 5-min rest between test activities (resting mandibular position, maximum voluntary clenching in intercuspal position, maximum voluntary clenching on dental cotton rolls in intercuspal position, pain free maximum unassisted opening)^46^. There were no significant differences (*p* > 0.05) between repeated sEMG recordings in all analyzed variables (Table 1). Automatic processing of the sEMG signal using the BioPAK system converted the signal to Root Mean Square (RMS). RMS was used to analyze muscle activity. The study was conducted by an experienced physiotherapist who specializes in electromyography (first author).

### Pressure pain thresholds examination

A standard FDX 50 digital algometer (Wagner Instruments, Greenwich, CT, USA) was used for the study. This model of algometer is recommended for pain threshold and pain tolerance. Pressure Pain Thresholds (PPT) was defined as the amount of force required to produce a pain sensation distinct from pressure or discomfort, or otherwise; the point at which pressure transitions to discomfort or pain^50^. The algometer consisted of a pressure gauge and a 1 cm^2^ rubber piston tip with a digital display of force in 0.01 kgf increments. It was calibrated before each test^51^.

**Figure 1:**
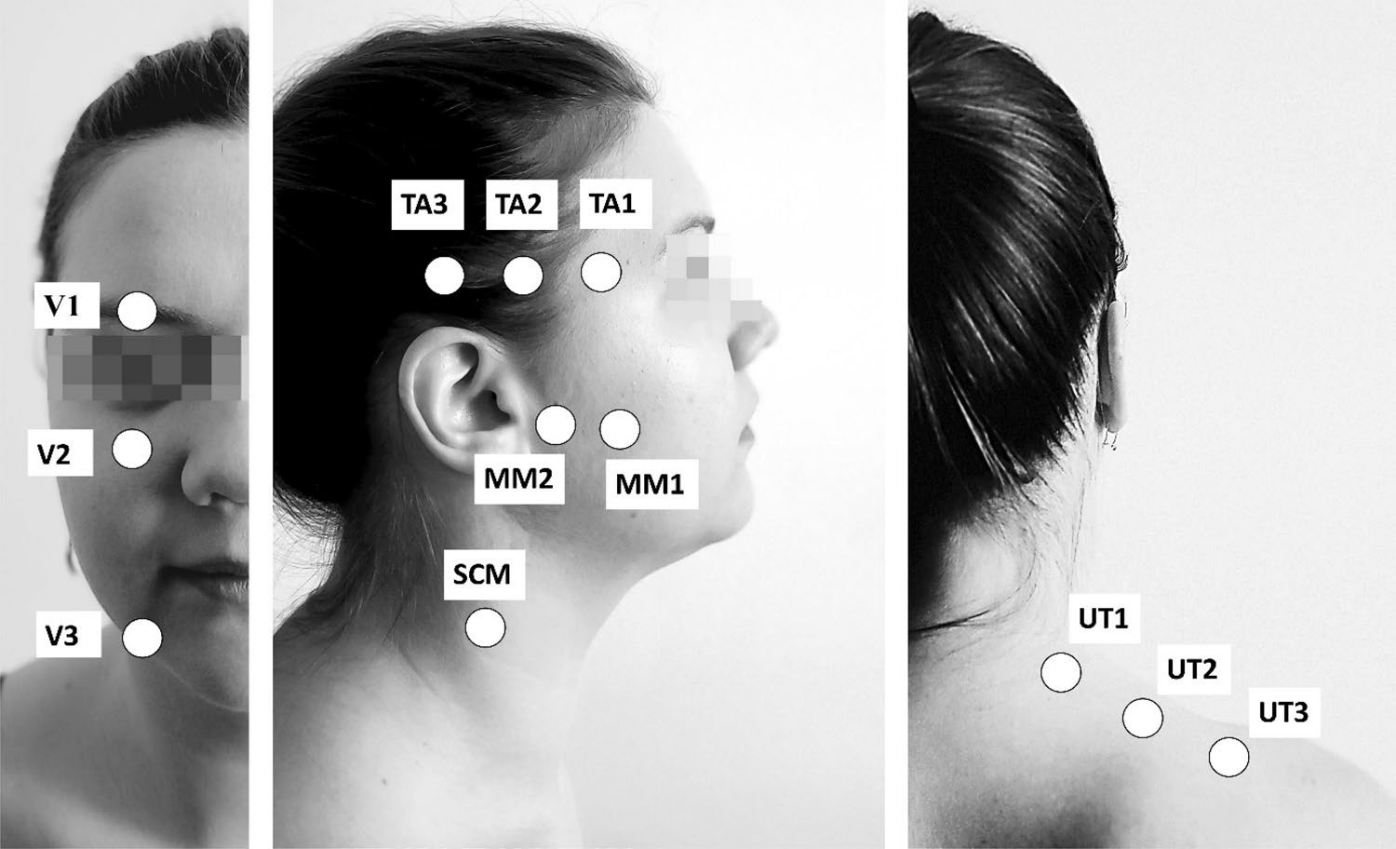
Tested points on Pressure Pain Thresholds. TA—the temporalis muscle; MM—the masseter muscle; SCM—the sternocleidomastoid muscle; DA—the digastric muscle; UT—the upper trapezius; V1— the output of the ophthalmic nerve; V2— the output of the maxillary nerve; V3— the output of the mandibular nerve;

The following points were tested:

- Trigeminal nerve outputs (the output of the ophthalmic nerve (supraorbital nerve output (V1)), the output of the maxillary nerve (infraorbital nerve output (V2)), the output of the mandibular nerve (chin nerve output (V3)))^52^;
- Two points on the masseter muscle (according to the Systematic Mapping of Pressure Pain Thresholds, two points were selected for MM—point number 7 (MM2) and point number 8 (MM1))^53^;
- Three points on the temporalis muscle (according to the Systematic Mapping of Pressure Pain Thresholds, three points were selected for TA—point number 13 (TA1), point number 10 (TA2) and point number 7 (TA3))^53^;
- One point on sternocleidomastoid muscle (in the middle course of the muscle at the height of C4 (SCM))^54^;
- Three points on the upper part of the trapezius muscle (points were determined in each subject in a straight line between the height of the seventh cervical vertebra and the lateral part of the shoulder process of the scapula, starting from the edge of the neck (UT 1) toward the shoulder process of the scapula at a distance of 2 cm from each other (UT2 and UT3))^55^ (Fig. 1).

The study was conducted by an experienced physiotherapist (first author).

### Ophthalmic examination

The patients were tested using a Snellen chart to examine the best-corrected visual acuity in the group with refractive error and to determine visual acuity in the group without refractive error^13, 56^.

The Topcon KR-800 autokeratorefractometer test (Topcon Co. Tokyo, Japan) was used to analyze the refractive error in the groups^57^.

IOL Master 500 equipment (Carl Zeiss Meditec, Jena, Germany) was used to determine eyeball length. Five separate measurements were taken and averaged for axial length^12, 13, 58^.

The thickness of the choroid and retina was performed by optical coherence tomography (OCT) (Optovue AngioVue (Fremont, CA, USA)). To avoid changes in choroidal and retinal thickness caused by time of day, the study was performed between 1 and 3 p.m. Analysis was performed in 6 × 6 mm scans centered on fovea. To ensure accuracy, the cutoff point for scan quality was 7/10^13^. The thickness of the choroid was measured manually using the built-in caliper in the OCT cross-sectional scans ^13, 59^.

Intraocular pressure testing was performed at the end of the examination to ensure that the administered anesthesia (ALCAINE 0.5% (Alcon Laboratories Inc., Fort Worth, TX, USA)) and any epithelial loss did not affect other measurements^13^. After administration of anesthesia, the Tono-Pen XL (Medtronic Solan, FL, USA) was positioned vertically to the anesthetized cornea. The averaged version from four outcomes was taken into consideration^13, 60^.

The ophthalmological examination was conducted by an experienced ophthalmologist (second author).

### Sleep quality assessment

The PSQI was used to assess sleep quality. It assesses the quality of sleep over the past month. It consists of a total of 19 items on which the respondent provides answers. The response portions are based on a 4-point Likert scale. The items from the PSQI are summed to obtain a total score measuring overall sleep quality. The total score for the PSQI scale ranges from 0 to 21 points, with a higher score indicating poorer sleep quality. A score above five indicates reduced quality^61^. It has high reliability (Cronbach’s alpha = 0.80)^62^.

### Statistical analysis

Statistical analysis was performed using Statistica software (version 13.3.721.1, StaSoft Poland TIBICO Software Inc. Palo Alto, CA, USA). An analysis of power was conducted using G*Power 3.1.9.7 (Heinrich-Heine-Universität Düsseldorf, Düsseldorf, Germany)^63^. It was calculated that a total sample size of 138 eyeballs (in 4 groups), would be sufficient to notice a significant difference in the ANOVA test with the assumptions of α value of 0.05, a power value of 0.90^64^, and an effect size of 0.40^65^. The Chi-square test was used to compare the number of females and males in groups. First, the normality of the distribution was verified using the Shapiro–Wilk test and the Kolmogorov–Smirnov test (with the Lillierfors correction). The Student’s t-test (T) or Mann–Whitney U test (Z) was used to compare the results of the sEMG repeatability tests, depending on the distribution. For the remaining analyses, it was decided to use non-parametric tests due to non-normal distributions. The Mann–Whitney U test was used to analyze between the two groups. Statistical significance in this test was set at *p* ≤ 0.05. The Kruskal–Wallis test (H) was used for analysis between four groups. With this test, a Bonferroni correction (alpha = 0.05/4 = 0.0125) was introduced, based on which the significance level was set at *p* ≤ 0.0125. The effect size for statistically significant results was calculated according to the formula (value obtained in the Kruskal–Wallis test—the number of groups + 1)/(the total number of observations—the number of groups). The effect size was defined as a small effect for 0.01– < 0.06, a moderate effect for 0.06–< 0.14, and a large effect for >  = 0.14^66^. A post hoc two-sided significance levels test with a Bonferroni adjustment was performed for statistically significant results. Spearman’s rank correlation coefficient was used for correlation analysis. The test varied between − 1 (perfect negative monotonic association) and + 1 (perfect positive monotonic association). Statistical significance in this test was set at *p* ≤ 0.05. A correlation was considered large for values greater than 0.5 and moderate for values between 0.3 and 0.5^67^.

Due to the number of performed analyses, the most important statistical results are presented below; a full description and complete analyses can be found in the supplementary material.

### Ethics approval

This study was conducted according to the Declaration of Helsinki principled. Approval was granted by the Medical University of Lublin Bio Ethics Committee (approval number KE-0254/229/2020).

## Results

Statistical comparisons of number, age and BMI showed no statistical differences between the groups. The groups with myopia did not differ in the size of the refractive error between them. Individuals without a refractive error had a visual acuity of 1.0 and those with a refractive error had the best corrected visual acuity of 1.0 as well. There were no differences between intraocular pressure, choroidal thickness and retinal thickness. Differences in maximum unassisted opening and maximum assisted opening retraction were shown between groups. Patients with Myopia (Without TMDs) showed the lowest maximum unassisted opening and maximum assisted opening of all groups (Table 2, Table 1 in the supplementary material 1).

**Table 2:** Presentation of groups.

	Myopia & TMDs		Myopia (without TMDs)		Emmetropic & TMDs		Emmetropic (without TMDs)			
	Mean	SD	Mean	SD	Mean	SD	Mean	SD	*p*	ES
n eyeballs	42	40	32	42						
n female eyeballs	34	38	24	24					*0.47*	
n male eyeballs	8	12	8	18						
age	24.63	2.69	24.00	2.49	23.88	2.33	23.86	2.31	*0.46*	
BMI	22.53	3.52	22.57	3.29	20.86	4.77	22.46	3.66	*0.81*	
Best corrected visual acuity										
R	1.0		1.0		n/a		n/a		*n/a*	
L	1.0		1.0		n/a		n/a		*n/a*	
Visual Acuity										
R	n/a		n/a		1.0		1.0		*n/a*	
L	n/a		n/a		1.0		1.0		*n/a*	
Refractive error (dsph)	− 2.50	1.25	− 2.00	1.00	n/a		n/a		*0.12*	
Intraocular pressure (mmHg)	13.76	4.52	14.58	3.98	13.29	3.88	13.64	3.91	*0.55*	
Retinal thickness (μm)	249.17	13.46	254.33	12.77	253.96	17.73	255.86	17.92	*0.15*	
Choroidal thickness (μm)	296.33	65.89	320.65	87.90	342.21	65.25	339.55	102.21	*0.10*	
Axial length (mm)	24.18	0.81	24.13	0.87	23.57	0.62	23.61	0.70	0.00*	***0.11***
Mandibular range of motion (mm)										
Pain free opening	45.05	10.53	45.60	6.82	45.31	7.29	49.70	7.02	*0.06*	
Maximum unassisted opening	49.55	6.20	45.85	7.07	49.25	5.20	49.95	7.04	0.01*	***0.05***
Maximum assisted opening	52.45	6.07	47.85	7.18	52.00	4.74	53.15	6.98	0.00*	***0.08***
Mandibular movement to the right	9.20	2.37	9.65	1.44	9.69	3.04	10.20	2.43	*0.73*	
Mandibular movement to the left	11.70	3.14	10.10	2.48	10.75	2.60	10.50	2.87	*0.07*	
Protrusion	7.05	2.04	6.40	2.06	7.44	2.38	7.30	2.70	*0.44*	

n, individuals in the sample; SD, standard deviation; BMI, body mass index; R, right side; L, left side; Dsph, spherical diopter; mmHg, conventional millimeters of mercury; μm, micrometer; mm, millimeter; ES, effect size; *significant difference.

Significant values are bold, italic.

After between-group differences in PSQI scores were shown, the Emmetropic & TMDs group had the highest scores, followed by the Myopia & TMDs group. Statistically significant differences in PPT on TA1-3, MM2, SCM, V2, and V3, in the Myopia & TMDs group were also shown. This group showed the lowest values of the tested PPT (Table 3, Table 5 in the supplementary material 1).

**Table 3:** Comparison of PSQI score, Pressure Pain Threshold, bioelectrical activity scores between groups.

		Myopia & TMDs		Myopia (without TMDs)		Emmetropic & TMDs		Emmetropic (without TMDs)			
		Mean	SD	Mean	SD	Mean	SD	Mean	SD	*p*	ES
PSQI score		5.62	2.70	4.10	1.97	8.06	3.34	4.43	2.68	0.00*	***0.17***
Pressure pain threshold	TA 1	2.06	0.93	2.99	1.34	2.47	0.98	2.95	1.05	0.00*	***0.08***
	TA 2	2.54	0.96	3.46	1.28	3.04	1.00	3.56	1.05	0.00*	***0.11***
	TA 3	2.85	1.01	3.89	1.21	3.32	0.95	3.81	1.12	0.00*	***0.11***
	MM 1	1.94	0.90	2.46	1.07	1.97	0.70	2.33	0.77	*0.03*	
	MM 2	1.97	0.91	2.73	1.09	2.04	0.69	2.51	0.75	0.00*	***0.04***
	SCM	1.37	0.78	1.86	1.01	1.77	1.23	1.77	0.64	0.00*	***0.11***
	UT 1	2.91	1.25	3.48	1.29	3.18	1.28	3.54	1.21	*0.02*	
	UT 2	3.40	1.17	3.93	1.14	3.55	1.22	3.91	1.19	*0.10*	
	UT 3	3.69	1.23	4.28	0.98	3.86	1.15	4.06	1.07	*0.06*	
	V1	2.60	1.14	3.25	1.55	3.10	1.15	3.78	1.12	*0.11*	
	V2	1.93	1.00	2.60	1.34	2.30	1.05	2.68	1.02	0.00*	***0.08***
	V3	2.11	0.93	2.90	1.30	2.47	1.08	2.99	0.87	0.01*	***0.06***
Resting mandibular position	TA	3.45	3.17	2.22	1.01	3.50	2.69	2.89	1.90	0.00*	***0.07***
	MM	2.27	1.72	2.49	1.82	2.27	1.30	2.31	1.26	*0.57*	
	SCM	1.41	0.66	1.29	0.44	1.24	0.43	1.28	0.43	*0.90*	
	DA	2.19	1.05	1.98	1.26	1.71	0.80	1.54	0.57	*0.87*	
The maximum voluntary clenching in intercuspal position	TA	111.10	71.29	133.98	59.35	154.18	70.95	126.38	66.24	*0.06*	
	MM	123.77	108.14	156.76	103.29	201.62	119.18	141.29	64.56	0.00*	***0.08***
	SCM	7.68	7.25	12.50	9.60	12.33	9.19	8.09	3.59	0.00*	***0.09***
	DA	20.69	12.97	23.55	15.49	18.26	6.92	16.10	9.51	*0.10*	
The maximum voluntary clenching on dental cotton rolls in intercuspal position	TA	136.33	179.33	120.58	50.44	126.39	66.74	122.52	69.42	*0.77*	
	MM	141.09	90.62	167.67	97.41	192.19	114.17	163.50	70.84	*0.11*	
	SCM	8.85	7.05	14.80	10.14	14.52	10.00	10.77	4.92	0.00*	***0.13***
	DA	20.15	9.45	25.64	13.76	21.55	7.75	19.80	9.81	*0.15*	
The pain free maximum unassisted opening	TA	8.24	5.61	11.51	12.18	6.37	2.85	9.61	9.52	*0.30*	
	MM	10.18	11.18	17.76	18.66	9.57	7.20	12.37	13.38	*0.05*	
	SCM	12.96	13.14	17.76	22.21	13.27	15.93	12.84	14.39	*0.50*	
	DA	75.25	34.26	99.61	46.70	92.91	61.72	60.75	33.85	0.00*	***0.07***

PSQI—the Pittsburgh Sleep Quality Index; SD—standard deviation; TA—the temporalis muscle; MM—the masseter muscle; SCM—the sternocleidomastoid muscle; DA—the digastric muscle; UT—the upper trapezius; V1— the output of the ophthalmic nerve; V2— the output of the maxillary nerve; V3— the output of the mandibular nerve; ES—effect size; *significant difference.

Significant values are bold, italic.

When analyzing bioelectrical activity, significant differences were observed during resting mandibular position on TA. The Emmetropic & TMDs group showed the highest values (Table 3, Table 5 in the supplementary material 1). Other statistically significant differences were observed in the maximum voluntary clenching in intercuspal position on MM and SCM. The Emmetropic & TMDs group showed the highest bioelectrical activities on MM, and the Myopia & TMDs group showed the lowest. On the SCM, the highest values were observed in the Myopia (Without TMDs) group and the lowest values in the Myopia & TMDs group. Differences were again observed on SCM in the maximum voluntary clenching on dental cotton rolls in intercuspal position, with the highest values shown by the Myopia (Without TMDs) group and the lowest by the Myopia & TMDs group. When analyzing the pain free maximum unassisted opening, differences were shown on DA, the Myopia (Without TMDs) group had the highest values, and the Emmetropic (Without TMDs) group had the lowest values (Table 3, Table 5 in the supplementary material 1).

Correlations were shown in refractive error and the maximum voluntary clenching in intercuspal position on SCM in the Myopia & TMDs group (positive correlations) and in the Myopia (Without TMDs) group (negative correlations) (Table 19 in the supplementary material 1). Correlations were shown in the intraocular pressure and in the the maximum voluntary clenching on dental cotton rolls in intercuspal position on DA in the Myopia (Without TMDs) group (positive correlations) and in the Emmetropic (Without TMDs) group (negative correlations) (Table 20 in the supplementary material 1). Correlations were shown in retinal thickness and during resting mandibular position on SCM in the Myopia & TMDs group (negative correlations) and in the Emmetropic (Without TMDs) group (positive correlations) (Table 21 in the supplementary material 1).

Most correlations were observed between choroidal thickness and PPT. In the Myopia (Without TMDs) group, positive correlations (high to medium correlation values) were observed on all tested points. In the Emmetropic & TMDs group, negative correlations were observed on the TA1 and TA2 points. In the Emmetropic (Without TMDs) group, positive correlations were observed on points TA1-3, UT1-3 and V1 (Table 4 and Table 22 in the supplementary material 1). Correlations were seen between choroidal thickness and bioelectrical activity during the pain free maximum unassisted opening on DA in the Myopia & TMDs group and Emmetropic (Without TMDs) group (negative correlations) and also in the Myopia (Without TMDs) group (positive correlations) (Table 22 in the supplementary material 1).

**Table 4:** Correlation results between choroidal thickness.

			Myopia & TMDs	Myopia (without TMDs)	Emmetropic & TMDs	Emmetropic without TMDs)
			*p*	*p*	*p*	*p*
Choroidal thickness (μm)	Pressure pain threshold	TA 1	*0.25*	0.00*	0.01*	0.00*
		TA 2	*0.84*	0.00*	0.04*	0.01*
		TA 3	*0.59*	0.00*	*0.13*	0.00*
		MM 1	*0.71*	0.00*	*0.29*	*0.34*
		MM 2	*0.69*	0.01*	*0.24*	*0.22*
		SCM	*0.89*	0.00*	*0.79*	*0.08*
		UT 1	*1.00*	0.00*	*0.24*	0.00*
		UT 2	*0.45*	0.01*	0.02*	0.02*
		UT 3	*0.93*	0.01*	*0.34*	0.01*
		V1	*0.41*	0.00*	*0.41*	0.02*
		V2	*0.24*	0.00*	*0.97*	*0.08*
		V3	*0.62*	0.00*	*0.68*	*0.15*

PSQI, the Pittsburgh Sleep Quality Index; TA, the temporalis muscle; MM, the masseter muscle; SCM, the sternocleidomastoid muscle; DA, the digastric muscle; UT, the upper trapezius; V1, the output of the ophthalmic nerve; V2, the output of the maxillary nerve; V3, the output of the mandibular nerve; μm, micrometer; *significant difference.

Significant values are bold, italic.

When analyzing the correlation between axial length and PSQI, PPT, and bioelectrical activity scores, the Myopia (Without TMDs) group showed negative correlations between PSQI, TA3, UT3. In the same group negative correlations were observed when analyzing bioelectrical activity in resting mandibular position on DA, positive correlations in the maximum voluntary clenching in intercuspal position on DA and in the maximum voluntary clenching on dental cotton rolls in intercuspal position on MM. No correlations were shown in the Myopia & TMDs group. Additionally, in the Emmetropic (Without TMDs) group, positive correlations were observed during PPT on TA1, MM2 and also positive correlations during the pain free maximum unassisted opening on MM, SCM and DA (Table 23 in the supplementary material 1).

Multiple correlations were observed between PSQI score and the maximum voluntary clenching in intercuspal position in the Emmetropic & TMDs group (negative correlations on TA, MM and SCM) and in the Emmetropic (Without TMDs) group (positive correlations on MM, DA). Further correlations were seen during the maximum voluntary clenching on dental cotton rolls in intercuspal position in the Emmetropic & TMDs group (negative correlations on SCM) and in the Emmetropic (Without TMDs) group (positive correlations on TA, MM and DA) (Table 5 and Table 24 in the supplementary material 1).

**Table 5:** Correlation results between PSQI score and bioelectrical activity scores between groups.

			Myopia & TMDs	Myopia (without TMDs)	Emmetropic & TMDs	Emmetropic (without TMDs)
			*p*	*p*	*p*	*p*
PSQI score	Resting mandibular position	TA	*0.86*	*0.62*	*0.69*	*0.81*
		MM	*0.67*	*0.07*	*0.90*	*0.40*
		SCM	*0.19*	*0.19*	*0.97*	*0.08*
		DA	*0.15*	*0.41*	*0.86*	*0.05*
	The maximum voluntary clenching in intercuspal position	TA	*0.37*	*0.86*	0.02*	*0.06*
		MM	*0.21*	*0.42*	0.00*	0.01*
		SCM	*0.68*	*0.42*	0.01*	*0.42*
		DA	*0.80*	*0.64*	*0.11*	0.04*
	The maximum voluntary clenching on dental cotton rolls in intercuspal position	TA	*0.90*	*0.86*	*0.07*	0.00*
		MM	*0.56*	*0.68*	*0.15*	0.00*
		SCM	*0.99*	*0.69*	0.01*	*0.74*
		DA	*0.52*	*0.72*	*0.77*	0.01*
	The pain free maximum unassisted opening	TA	*0.13*	*0.72*	*0.76*	*0.06*
		MM	*0.61*	*0.24*	*0.36*	*0.24*
		SCM	*0.64*	*0.16*	*0.05*	*0.34*
		DA	*0.27*	*0.43*	*0.29*	0.02*

PSQI, the Pittsburgh Sleep Quality Index; TA, the temporalis muscle; MM, the masseter muscle; SCM, the sternocleidomastoid muscle; DA, the digastric muscle; *significant difference.

Significant values are bold, italic.

When analyzing mandibular movements, negative correlations were observed between intraocular pressure and during mandibular movement to the right and left in the Emmetropic (Without TMDs) group (Table 26 in the supplementary material 1). Further negative correlations were observed during the same movements and choroidal thickness in the Emmetropic & TMDs group (Table 28 in the supplementary material 1). The highest number of correlations were observed between PSQI score and all studied mandibular movements (positive correlations ranging from medium to high) in the Emmetropic (Without TMDs) group (Table 6, Table 30 in the supplementary material 1).

**Table 6:** Correlation results between PSQI score and mandibular range of motion.

			Myopia & TMDs	Myopia (without TMDs)	Emmetropic & TMDs	Emmetropic (without TMDs)
			*p*	*p*	*p*	*p*
PSQI score	Mandibular range of motion (mm)	Pain free opening	0.01*	*0.79*	*0.38*	0.00*
		Maximum unassisted opening	*0.17*	*0.62*	0.03*	0.00*
		Maximum assisted opening	*0.32*	*0.68*	*0.11*	0.00*
		Mandibular movement to the right	*0.41*	*0.29*	*0.91*	0.00*
		Mandibular movement to the left	*0.14*	*0.34*	*0.55*	0.02*
		Protrusion	*0.11*	*0.85*	*0.13*	0.00*

PSQI, the Pittsburgh Sleep Quality Index; mm, millimeter; *significant difference.

Significant values are bold, italic.

## Discussion

To date, no study has differentiated the reciprocal effects of myopia and TMDs on PPT, muscular system, electromyographic activity and sleep quality. These disease entities have not been viewed as influencing each other. The main aim of this study is to evaluate the effects of TMDs and myopia on the connections between the visual organ, the bioelectrical activity and sensitivity of the masticatory and cervical muscles, and sleep quality. Another aim is to develop additional guidelines for electromyographic examination of patients with ophthalmic problems. To the best of the author’s knowledge, this is the first study of its kind.

Based on the results, it was observed that the group that achieved the highest scores on the PSQI questionnaire was the Emmetropic & TMDs group. The analysis by Lee et al. showed that patients with TMDs have poorer sleep quality compared to healthy subjects^19^. The refractive error was not taken into account by the author of the description. In second place in terms of PSQI score is the Myopia & TMDs group. It is worth noting that in this study, the authors analyzed subjects with low myopia^30^, which may have played a role in this result.

Also, the lowest PPT scores were noted in the Myopia & TMDs group. In addition to the connections between the systems, this shows the hypersensitivity of the tested muscles in this group and the merging of the two disfunction entities. Supposedly, this could be related to central nervous system sensitization caused by the two entities^68, 69^.

There were also differences in the bioelectrical activity of the masticatory muscles: in resting mandibular position on TA, in the maximum voluntary clenching in intercuspal position on MM and SCM, in the maximum voluntary clenching on dental cotton rolls in intercuspal position on SCM and in the pain free maximum unassisted opening on DA. At resting mandibular position, subjects in the Emmetropic & TMDs group showed the highest bioelectrical tension on the TA muscles. The Emmetropic & TMDs group showed the highest bioelectrical activities on MM, and the Myopia & TMDs group showed (on MM and SCM) the lowest in the maximum voluntary clenching in intercuspal position. In this study, the authors did not examine the change in visual input (open and closed eye test)^14–16, 42^, this was the activity observed with eyes open without correction (glasses and lenses). Connections between systems may be due to components of several systems, for example, neural or fascial^11, 13^. To date, electromyographic studies on the masticatory muscles have not marked or differentiated into refractive error and emmetropic subjects^70–72^. The fact is that this also affects the electromyographic record and thus also affects the results and possible conclusions in the works. Therefore, the authors of this research suggest and recommend:

- The mandatory first information in the research in the methods section how many subjects were with a refractive error and the size of the refractive error.
- Possibly exclude people with a refractive error from sEMG studies if the researcher wants to ideally determine the effect factor of another disease entity on the muscles or the selection of the same number of people with the same refractive error in the control group.
- If none of the recommendations have been done in work—information about it in the limitations paragraph.

A case study involving a change in the correction of a refractive error are able to affect muscle activity in a short period of time^73^. This supports the observations made by the authors of this research about the direct effect on the recording of muscle bioelectrical activity and the need to mark it.

Correlations were observed between refractive error and bioelectrical activity in the maximum voluntary clenching in intercuspal position (Table 19 in the supplementary material 1) in the Myopia & TMDs and Myopia (Without TMDs) groups. It is worth noting the differences in correlations—positive correlations were observed in the Myopia & TMDs group and negative correlations in the Myopia (Without TMDs) group. The current study confirms the connections between the cervical segment and the TMDs^74^, and also between the cervical segment and myopia^75^. Further research into the observed inverse correlation is required.

A noteworthy correlation is between choroidal thickness and PPT. Positive correlations were observed on all tested points in the Myopia (Without TMDs) group. In the Emmetropic (Without TMDs) group, positive correlations were also observed on points TA1-3, UT1-3 and V1 (Table 4 and Table 22 in the supplementary material 1). Again, this demonstrates a link between the systems. The lack of results in the groups with TMDs shows that, hypothetically, there are changes in the connections between the systems. It is suggested, that changes in choroidal thickness are also correlated with changes in the growth of the sclera and the eye itself^76^. The Nickla and Wallman research explain that choroidal thickening may be mechanically linked to the synthesis of scleral macromolecules, and this may be associated with homeostatic control of growth (consequently accounting for the etiology of myopia and hyperopia)^76^. Explaining the connections that may play a key role here are the connections through the fascial network pathway (Tenon’s capsule—upper eyelid elevator, orbicularis oculi, sequentially superficial musculoaponeurotic system^13^) or the connection through the neurological pathway (cranial nerves and in particular—II optic; III oculomotor; IV trochlear; V trigeminal; VI abducent; XI accessory^11, 77^).

Another correlation to be noted in this discussion is axial length versus PSQI score. A negative correlation was observed in the Myopia (Without TMDs) group. The problem of sleep quality and myopia is recognized in the research^18^, but the researchers are not noting that sleep duration was related to myopia and eyeball axial length^78^, which is consistent with an observable negative correlation (Table 23 in the supplementary material 1). In this group, there were correlations between PPT and bioelectrical activity. No, correlations were observed in the Myopia & TMDs group. In the Emmetropic (Without TMDs) group, positive correlations were observed during pain free maximum unassisted opening on MM, SCM, and DA (Table 23 in the supplementary material 1). However, this phenomenon has not been observed in previous studies^13, 12^. The phenomenon requires further study.

As for correlations between PSQI scores and bioelectrical activity, such were observed in the Emmetropic (Without TMDs) and Emmetropic & TMDs groups. These were correlations on various muscles, except MM in the maximum voluntary clenching in intercuspal position. It is worth noting that there was a negative correlation here in the group with TMDs and a positive correlation in the group without TMDs (Table 5 and Table 24 in the supplementary material 1). A possible explanation for this phenomenon is, not studied by us, bruxism activity during sleep. Additionally, the PSQI score was associated with mandibular mobility. Research suggests that bruxism is associated with greater masseter muscle activity^79^. Clenching the jaw during bruxism can cause acute tenderness^80^ and affect sleep quality.

This study contains the following limitations. The refractive error is related to the person’s race, so the authors of this research suggest testing on other races^81^. Next, the diagnostic criteria for TMDs were changed to The Diagnostic Criteria for Temporomandibular Disorders (DC/TMD) in 2014^82^. However, the present study used the previous version. To date, there is no validated Polish version of DC/TMD, so RDC/TMD was used. In this study, patients with correction (glasses, lenses) were not examined because the authors did not know how this might affect the bioelectrical activity and PPT. This was dictated by two factors: the lack of information on the effect of metal corrective frames on sEMG recording, and the lenses would have prevented the study of intraocular pressure (application of ALCAINE 0.5%). In terms of the strengths of the study, to the authors’ knowledge it was the first of its kind in the world. It was carried out in a large group of people. The effect size of the statistically significant results was in the range of moderate to large. Given the observed effects of refractive error and TMDs on the musculoskeletal system and sleep quality, further research in this area is suggested to clarify possible anatomical relationships.

## Conclusions

- The organ of vision is connected to the masticatory and the cervical segment muscles.
- TMDs and myopia affect the resting and functional activity of the masticatory and cervical muscles.
- The thickness of the choroid in individuals with myopia is associated with the temporalis, the masseter, and cervical muscles (sternocleidomastoid muscle and upper trapezius muscle).
- TMDs and myopia impair sleep quality.
- Sleep quality is associated with a mandibular range of motion.
- It is recommended to compare bioelectrical activity between different groups only with the same number of people with refractive error. And, above all, the exact number of refractive errors and the number of subjects with this error in the study should be specified in the methodology section.

### Supplementary Information

Supplementary Tables.

## Data Availability

The datasets generated during and/or analyzed during the current study are available from the corresponding author on reasonable request.
