# Supplementary material for: Myopia & painful muscle form of temporomandibular disorders: connections between vision, masticatory and cervical muscles activity and sensitivity and sleep quality
Source: Sci Rep. 2023 Nov 19;13:20231. doi: 10.1038/s41598-023-47550-6 (PMC10658172; doi:10.1038/s41598-023-47550-6)
Supplement: Supplementary file 1 — Supplementary Tables. [file 41598_2023_47550_MOESM1_ESM.docx]

Table of contents

[Table 1. Presentation of groups. 3](#_Toc149135974)

[Table 2. Post hoc analysis for axial length. 4](#_Toc149135975)

[Table 3. Post hoc analysis for maximum unassisted opening. 4](#_Toc149135976)

[Table 4. Post hoc analysis for maximum assisted opening. 4](#_Toc149135977)

[Table 5. Comparison of PSQI, pressure pain threshold, bioelectrical activity scores between groups. 5](#_Toc149135978)

[Table 6. Post hoc analysis for PSQI score. 6](#_Toc149135979)

[Table 7. Post hoc analysis for pressure pain threshold (TA 1). 6](#_Toc149135980)

[Table 8. Post hoc analysis for pressure pain threshold (TA 2). 7](#_Toc149135981)

[Table 9. Post hoc analysis for pressure pain threshold (TA 3). 7](#_Toc149135982)

[Table 10. Post hoc analysis for pressure pain threshold (MM 2). 7](#_Toc149135983)

[Table 11. Post hoc analysis for pressure pain threshold (SCM). 7](#_Toc149135984)

[Table 12. Post hoc analysis for pressure pain threshold (V2). 8](#_Toc149135985)

[Table 13. Post hoc analysis for pressure pain threshold (V3). 8](#_Toc149135987)

[Table 14. Post hoc analysis for bioelectrical activity in resting mandibular position (TA). 8](#_Toc149135988)

[Table 15. Post hoc analysis for bioelectrical activity in the maximum voluntary clenching in intercuspal position (MM). 9](#_Toc149135989)

[Table 16. Post hoc analysis for bioelectrical activity in the maximum voluntary clenching in intercuspal position (SCM). 9](#_Toc149135990)

[Table 17. Post hoc analysis for bioelectrical activity in the maximum voluntary clenching on dental cotton rolls in intercuspal position (SCM). 9](#_Toc149135992)

[Table 18. Post hoc analysis for bioelectrical activity in the pain free maximum unassisted opening (DA). 10](#_Toc149135993)

[Table 19. Correlation results between refractive error and PSQI score, pressure pain threshold, bioelectrical activity scores between groups. 11](#_Toc149135994)

[Table 20. Correlation results between intraocular pressure and PSQI score, pressure pain threshold, bioelectrical activity scores between groups. 12](#_Toc149135995)

[Table 21. Correlation results between retinal thickness and PSQI score, pressure pain threshold, bioelectrical activity scores between groups. 13](#_Toc149135996)

[Table 22. Correlation results between choroidal thickness and PSQI score, pressure pain threshold, bioelectrical activity scores between groups. 14](#_Toc149135997)

[Table 23. Correlation results between axial length and PSQI score, pressure pain threshold, bioelectrical activity scores between groups. 15](#_Toc149135998)

[Table 24. Correlation results between PSQI score and pressure pain threshold, bioelectrical activity scores between groups. 16](#_Toc149135999)

[Table 25. Correlation results between refractive error and mandibular range of motion. 17](#_Toc149136000)

[Table 26. Correlation results between intraocular pressure and mandibular range of motion. 17](#_Toc149136001)

[Table 27. Correlation results between retinal thickness an mandibular range of motion. 17](#_Toc149136002)

[Table 28. Correlation results between choroidal thickness and mandibular range of motion. 18](#_Toc149136003)

[Table 29. Correlation results between axial length and mandibular range of motion. 18](#_Toc149136004)

[Table 30. Correlation results between PSQI score and mandibular range of motion. 18](#_Toc149136005)

# Table 1. Presentation of groups.

|  |  | | **Myopia & TMDs** | | **Myopia**  **(Without TMDs)** | | **Emmetropic & TMDs** | | **Emmetropic**  **(Without TMDs)** | |  |  |  |  |
| --- | --- | --- | --- | --- | --- | --- | --- | --- | --- | --- | --- | --- | --- | --- |
|  |  | | **Mean** | **SD** | **Mean** | **SD** | **Mean** | **SD** | **Mean** | **SD** | **test** |  | **p** | **ES** |
|  | **n eyeballs** | | 42 | | 40 | | 32 | | 42 | |  |  |  |  |
|  | **n female eyeballs** | | 34 | | 38 | | 24 | | 24 | | X^2^ | 2.55 | *0.47* |  |
|  | **n male eyeballs** | | 8 | | 12 | | 8 | | 18 | |  |  |  |  |
|  | **age** | | 24.63 | 2.69 | 24.00 | 2.49 | 23.88 | 2.33 | 23.86 | 2.31 | H | 2.59 | *0.46* |  |
|  | **BMI** | | 22.53 | 3.52 | 22.57 | 3.29 | 20.86 | 4.77 | 22.46 | 3.66 | H | 0.97 | *0.81* |  |
|  | **Best Corrected Visual Acuity** | **R** | 1.0 |  | 1.0 |  | n/a |  | n/a |  |  |  | *n/a* |  |
|  |  | **L** | 1.0 |  | 1.0 |  | n/a |  | n/a |  |  |  | *n/a* |  |
|  | **Visual Acuity** | **R** | n/a |  | n/a |  | 1.0 |  | 1.0 |  |  |  | *n/a* |  |
|  |  | **L** | n/a |  | n/a |  | 1.0 |  | 1.0 |  |  |  | *n/a* |  |
|  | **Refractive Error (Dsph)** | | -2.50 | 1.25 | -2.00 | 1.00 | n/a |  | n/a |  | Z | -1.57 | *0.12* |  |
|  | **Intraocular Pressure (mmHg)** | | 13.76 | 4.52 | 14.58 | 3.98 | 13.29 | 3.88 | 13.64 | 3.91 | H | 2.12 | *0.55* |  |
|  | **Retinal Thickness (μm)** | | 249.17 | 13.46 | 254.33 | 12.77 | 253.96 | 17.73 | 255.86 | 17.92 | H | 5.30 | *0.15* |  |
|  | **Choroidal Thickness (μm)** | | 296.33 | 65.89 | 320.65 | 87.90 | 342.21 | 65.25 | 339.55 | 102.21 | H | 6.22 | *0.10* |  |
|  | **Axial Length (mm)** | | 24.18 | 0.81 | 24.13 | 0.87 | 23.57 | 0.62 | 23.61 | 0.70 | H | 18.99 | ***0.00**** | ***0.11*** |
| **Mandibular Range Of Motion (mm)** | **Pain Free Opening** | | 45.05 | 10.53 | 45.60 | 6.82 | 45.31 | 7.29 | 49.70 | 7.02 | H | 7.31 | *0.06* |  |
|  | **Maximum Unassisted Opening** | | 49.55 | 6.20 | 45.85 | 7.07 | 49.25 | 5.20 | 49.95 | 7.04 | H | 10.80 | ***0.01**** | ***0.05*** |
|  | **Maximum Assisted Opening** | | 52.45 | 6.07 | 47.85 | 7.18 | 52.00 | 4.74 | 53.15 | 6.98 | H | 15.80 | ***0.00**** | ***0.08*** |
|  | **Mandibular Movement to The Right** | | 9.20 | 2.37 | 9.65 | 1.44 | 9.69 | 3.04 | 10.20 | 2.43 | H | 1.29 | *0.73* |  |
|  | **Mandibular Movement to The Left** | | 11.70 | 3.14 | 10.10 | 2.48 | 10.75 | 2.60 | 10.50 | 2.87 | H | 7.00 | *0.07* |  |
|  | **Protrusion** | | 7.05 | 2.04 | 6.40 | 2.06 | 7.44 | 2.38 | 7.30 | 2.70 | H | 2.72 | *0.44* |  |

n—individuals in the sample; SD—standard deviation; BMI—body mass index; R—right side; L—left side; Dsph—spherical diopter; mmHg—conventional millimeters of mercury; μm—micrometer; mm—millimeter; ES—effect size; x^2^—the Chi-square test; Z— the Mann-Whitney U test; H—the Kruskal-Wallis test; * significant difference.

# Table 2. Post hoc analysis for axial length.

| **Axial Length (mm)** | **Myopia & TMDs** | **Myopia**  **(Without TMDs)** | **Emmetropic & TMDs** | **Emmetropic**  **(Without TMDs)** |
| --- | --- | --- | --- | --- |
| **Myopia & TMDs** |  | *1.000000* | *0.085639* | ***0.017269**** |
| **Myopia (Without TMDs)** | *1.000000* |  | *0.091266* | ***0.019522**** |
| **Emmetropic & TMDs** | *0.085639* | *0.091266* |  | *1.000000* |
| **Emmetropic(Without TMDs)** | ***0.017269**** | ***0.019522**** | *1.000000* |  |

mm—millimeter; * significant difference.

# Table 3. Post hoc analysis for maximum unassisted opening.

| **Maximum Unassisted Opening (mm)** | **Myopia & TMDs** | **Myopia**  **(Without TMDs)** | **Emmetropic & TMDs** | **Emmetropic**  **(Without TMDs)** |
| --- | --- | --- | --- | --- |
| **Myopia & TMDs** |  | ***0.026272**** | *1.000000* | *1.000000* |
| **Myopia (Without TMDs)** | ***0.026272**** |  | *0.096664* | *0.050307* |
| **Emmetropic & TMDs** | *1.000000* | *0.096664* |  | *1.000000* |
| **Emmetropic(Without TMDs)** | *1.000000* | *0.050307* | *1.000000* |  |

mm—millimeter; * significant difference.

# Table 4. Post hoc analysis for maximum assisted opening.

| **Maximum Assisted Opening**  **(mm)** | **Myopia & TMDs** | **Myopia**  **(Without TMDs)** | **Emmetropic & TMDs** | **Emmetropic**  **(Without TMDs)** |
| --- | --- | --- | --- | --- |
| **Myopia & TMDs** |  | ***0.003016**** | *1.000000* | *1.000000* |
| **Myopia (Without TMDs)** | ***0.003016**** |  | ***0.040749**** | ***0.006313**** |
| **Emmetropic & TMDs** | *1.000000* | ***0.040749**** |  | *1.000000* |
| **Emmetropic(Without TMDs)** | *1.000000* | ***0.006313**** | *1.000000* |  |

mm—millimeter; * significant difference.

# Table 5. Comparison of PSQI, pressure pain threshold, bioelectrical activity scores between groups.

|  |  | **Myopia & TMDs** | | **Myopia (Without TMDs)** | | **Emmetropic & TMDs** | | **Emmetropic (Without TMDs)** | |  |  |  |
| --- | --- | --- | --- | --- | --- | --- | --- | --- | --- | --- | --- | --- |
|  |  | **Mean** | **SD** | **Mean** | **SD** | **Mean** | **SD** | **Mean** | **SD** | **test H** | **p** | **ES** |
| **PSQI score** | | 5.62 | 2.70 | 4.10 | 1.97 | 8.06 | 3.34 | 4.43 | 2.68 | 28.54 | ***0.00**** | ***0.17*** |
| **Pressure**  **Pain**  **Threshold** | TA 1 | 2.06 | 0.93 | 2.99 | 1.34 | 2.47 | 0.98 | 2.95 | 1.05 | 15.07 | ***0.00**** | ***0.08*** |
|  | TA 2 | 2.54 | 0.96 | 3.46 | 1.28 | 3.04 | 1.00 | 3.56 | 1.05 | 19.07 | ***0.00**** | ***0.11*** |
|  | TA 3 | 2.85 | 1.01 | 3.89 | 1.21 | 3.32 | 0.95 | 3.81 | 1.12 | 19.56 | ***0.00**** | ***0.11*** |
|  | MM 1 | 1.94 | 0.90 | 2.46 | 1.07 | 1.97 | 0.70 | 2.33 | 0.77 | 20.26 | *0.03* |  |
|  | MM 2 | 1.97 | 0.91 | 2.73 | 1.09 | 2.04 | 0.69 | 2.51 | 0.75 | 9.17 | ***0.00**** | ***0.04*** |
|  | SCM | 1.37 | 0.78 | 1.86 | 1.01 | 1.77 | 1.23 | 1.77 | 0.64 | 20.11 | ***0.00**** | ***0.11*** |
|  | UT 1 | 2.91 | 1.25 | 3.48 | 1.29 | 3.18 | 1.28 | 3.54 | 1.21 | 9.90 | *0.02* |  |
|  | UT 2 | 3.40 | 1.17 | 3.93 | 1.14 | 3.55 | 1.22 | 3.91 | 1.19 | 6.28 | *0.10* |  |
|  | UT 3 | 3.69 | 1.23 | 4.28 | 0.98 | 3.86 | 1.15 | 4.06 | 1.07 | 7.26 | *0.06* |  |
|  | V1 | 2.60 | 1.14 | 3.25 | 1.55 | 3.10 | 1.15 | 3.78 | 1.12 | 6.00 | *0.11* |  |
|  | V2 | 1.93 | 1.00 | 2.60 | 1.34 | 2.30 | 1.05 | 2.68 | 1.02 | 15.91 | ***0.00**** | ***0.08*** |
|  | V3 | 2.11 | 0.93 | 2.90 | 1.30 | 2.47 | 1.08 | 2.99 | 0.87 | 12.62 | ***0.01**** | ***0.06*** |
| **Resting**  **Mandibular**  **Position** | TA | 3.45 | 3.17 | 2.22 | 1.01 | 3.50 | 2.69 | 2.89 | 1.90 | 13.68 | ***0.00**** | ***0.07*** |
|  | MM | 2.27 | 1.72 | 2.49 | 1.82 | 2.27 | 1.30 | 2.31 | 1.26 | 2.03 | *0.57* |  |
|  | SCM | 1.41 | 0.66 | 1.29 | 0.44 | 1.24 | 0.43 | 1.28 | 0.43 | 0.57 | *0.90* |  |
|  | DA | 2.19 | 1.05 | 1.98 | 1.26 | 1.71 | 0.80 | 1.54 | 0.57 | 0.72 | *0.87* |  |
| **The Maximum**  **Voluntary**  **Clenching**  **in Intercuspal Position** | TA | 111.10 | 71.29 | 133.98 | 59.35 | 154.18 | 70.95 | 126.38 | 66.24 | 7.35 | *0.06* |  |
|  | MM | 123.77 | 108.14 | 156.76 | 103.29 | 201.62 | 119.18 | 141.29 | 64.56 | 15.74 | ***0.00**** | ***0.08*** |
|  | SCM | 7.68 | 7.25 | 12.50 | 9.60 | 12.33 | 9.19 | 8.09 | 3.59 | 17.18 | ***0.00**** | ***0.09*** |
|  | DA | 20.69 | 12.97 | 23.55 | 15.49 | 18.26 | 6.92 | 16.10 | 9.51 | 6.19 | *0.10* |  |
| **The Maximum**  **Voluntary**  **Clenching**  **on Dental Cotton Rolls**  **in Intercuspal Position** | TA | 136.33 | 179.33 | 120.58 | 50.44 | 126.39 | 66.74 | 122.52 | 69.42 | 1.14 | *0.77* |  |
|  | MM | 141.09 | 90.62 | 167.67 | 97.41 | 192.19 | 114.17 | 163.50 | 70.84 | 5.95 | *0.11* |  |
|  | SCM | 8.85 | 7.05 | 14.80 | 10.14 | 14.52 | 10.00 | 10.77 | 4.92 | 22.22 | ***0.00**** | ***0.13*** |
|  | DA | 20.15 | 9.45 | 25.64 | 13.76 | 21.55 | 7.75 | 19.80 | 9.81 | 5.31 | *0.15* |  |
| **The Pain Free**  **Maximum**  **Unassisted Opening** | TA | 8.24 | 5.61 | 11.51 | 12.18 | 6.37 | 2.85 | 9.61 | 9.52 | 3.65 | *0.30* |  |
|  | MM | 10.18 | 11.18 | 17.76 | 18.66 | 9.57 | 7.20 | 12.37 | 13.38 | 7.93 | *0.05* |  |
|  | SCM | 12.96 | 13.14 | 17.76 | 22.21 | 13.27 | 15.93 | 12.84 | 14.39 | 2.39 | *0.50* |  |
|  | DA | 75.25 | 34.26 | 99.61 | 46.70 | 92.91 | 61.72 | 60.75 | 33.85 | 13.30 | ***0.00**** | ***0.07*** |

PSQI—the Pittsburgh Sleep Quality Index; TA—the temporalis muscle; MM—the masseter muscle; SCM—the sternocleidomastoid muscle; DA—the digastric muscle; UT—the upper trapezius; V1— the output of the ophthalmic nerve; V2— the output of the maxillary nerve; V3— the output of the mandibular nerve; H—the Kruskal-Wallis test; ES—effect size; * significant difference.

# Table 6. Post hoc analysis for PSQI score.

| **PSQI score** | **Myopia & TMDs** | **Myopia**  **(Without TMDs)** | **Emmetropic & TMDs** | **Emmetropic**  **(Without TMDs)** |
| --- | --- | --- | --- | --- |
| **Myopia & TMDs** |  | *0.151208* | ***0.030641**** | *0.518362* |
| **Myopia (Without TMDs)** | *0.151208* |  | ***0.000007**** | *1.000000* |
| **Emmetropic & TMDs** | ***0.030641**** | ***0.000007**** |  | ***0.000066**** |
| **Emmetropic(Without TMDs)** | *0.518362* | *1.000000* | ***0.000066**** |  |

PSQI—the Pittsburgh Sleep Quality Index; * significant difference.

# Table 7. Post hoc analysis for pressure pain threshold (TA 1).

| **TA 1** | **Myopia & TMDs** | **Myopia**  **(Without TMDs)** | **Emmetropic & TMDs** | **Emmetropic**  **(Without TMDs)** |
| --- | --- | --- | --- | --- |
| **Myopia & TMDs** |  | ***0.002621**** | *0.456447* | ***0.000561**** |
| **Myopia (Without TMDs)** | ***0.002621**** |  | *0.731502* | *1.000000* |
| **Emmetropic & TMDs** | *0.456447* | *0.731502* |  | *0.377831* |
| **Emmetropic(Without TMDs)** | ***0.000561**** | *1.000000* | *0.377831* |  |

TA 1— first point of the temporalis muscle; * significant difference.

# Table 8. Post hoc analysis for pressure pain threshold (TA 2).

| **TA 2** | **Myopia & TMDs** | **Myopia**  **(Without TMDs)** | **Emmetropic & TMDs** | **Emmetropic**  **(Without TMDs)** |
| --- | --- | --- | --- | --- |
| **Myopia & TMDs** |  | ***0.003580**** | *0.220921* | ***0.000268**** |
| **Myopia (Without TMDs)** | ***0.003580**** |  | *1.000000* | *1.000000* |
| **Emmetropic & TMDs** | *0.220921* | *1.000000* |  | *0.525553* |
| **Emmetropic(Without TMDs)** | ***0.000268**** | *1.000000* | *0.525553* |  |

TA 2— second point of the temporalis muscle; * significant difference.

# Table 9. Post hoc analysis for pressure pain threshold (TA 3).

| **TA 3** | **Myopia & TMDs** | **Myopia**  **(Without TMDs)** | **Emmetropic & TMDs** | **Emmetropic**  **(Without TMDs)** |
| --- | --- | --- | --- | --- |
| **Myopia & TMDs** |  | ***0.000492**** | *0.695481* | ***0.001722**** |
| **Myopia (Without TMDs)** | ***0.000492**** |  | *0.195217* | *1.000000* |
| **Emmetropic & TMDs** | *0.695481* | *0.195217* |  | *0.430535* |
| **Emmetropic(Without TMDs)** | ***0.001722**** | *1.000000* | *0.430535* |  |

TA 3—third point of the temporalis muscle; * significant difference.

# Table 10. Post hoc analysis for pressure pain threshold (MM 2).

| **MM 2** | **Myopia & TMDs** | **Myopia**  **(Without TMDs)** | **Emmetropic & TMDs** | **Emmetropic**  **(Without TMDs)** |
| --- | --- | --- | --- | --- |
| **Myopia & TMDs** |  | ***0.001769**** | *1.000000* | ***0.007772**** |
| **Myopia (Without TMDs)** | ***0.001769**** |  | ***0.015093**** | *1.000000* |
| **Emmetropic & TMDs** | *1.000000* | ***0.015093**** |  | *0.051617* |
| **Emmetropic(Without TMDs)** | ***0.007772**** | *1.000000* | *0.051617* |  |

MM 2— second point of the masseter muscle; * significant difference.

# Table 11. Post hoc analysis for pressure pain threshold (SCM).

| **SCM** | **Myopia & TMDs** | **Myopia**  **(Without TMDs)** | **Emmetropic & TMDs** | **Emmetropic**  **(Without TMDs)** |
| --- | --- | --- | --- | --- |
| **Myopia & TMDs** |  | *0.137230* | *1.000000* | ***0.022116**** |
| **Myopia (Without TMDs)** | *0.137230* |  | *1.000000* | *1.000000* |
| **Emmetropic & TMDs** | *1.000000* | *1.000000* |  | *0.546043* |
| **Emmetropic(Without TMDs)** | ***0.022116**** | *1.000000* | *0.546043* |  |

SCM—the sternocleidomastoid muscle; * significant difference.

# Table 12. Post hoc analysis for pressure pain threshold (V2).

| **V2** | **Myopia & TMDs** | **Myopia**  **(Without TMDs)** | **Emmetropic & TMDs** | **Emmetropic**  **(Without TMDs)** |
| --- | --- | --- | --- | --- |
| **Myopia & TMDs** |  | *0.189490* | *0.611569* | ***0.000360**** |
| **Myopia (Without TMDs)** | *0.189490* |  | *1.000000* | *0.469052* |
| **Emmetropic & TMDs** | *0.611569* | *1.000000* |  | *0.255701* |
| **Emmetropic(Without TMDs)** | **0.000360*** | 0.469052 | 0.255701 |  |

# V2— the output of maxillary nerve; * significant difference.

# Table 13. Post hoc analysis for pressure pain threshold (V3).

| **V3** | **Myopia & TMDs** | **Myopia**  **(Without TMDs)** | **Emmetropic & TMDs** | **Emmetropic**  **(Without TMDs)** |
| --- | --- | --- | --- | --- |
| **Myopia & TMDs** |  | *0.083490* | *0.773172* | ***0.002956**** |
| **Myopia (Without TMDs)** | *0.083490* |  | *1.000000* | *1.000000* |
| **Emmetropic & TMDs** | *0.773172* | *1.000000* |  | *0.578474* |
| **Emmetropic(Without TMDs)** | ***0.002956**** | *1.000000* | *0.578474* |  |

V3— the output of mandibular nerve; * significant difference.

# Table 14. Post hoc analysis for bioelectrical activity in resting mandibular position (TA).

| **Resting Mandibular Position TA** | **Myopia & TMDs** | **Myopia**  **(Without TMDs)** | **Emmetropic & TMDs** | **Emmetropic**  **(Without TMDs)** |
| --- | --- | --- | --- | --- |
| **Myopia & TMDs** |  | ***0.014388**** | *1.000000* | ***0.000131**** |
| **Myopia (Without TMDs)** | ***0.014388**** |  | *0.812839* | *1.000000* |
| **Emmetropic & TMDs** | *1.000000* | *0.812839* |  | *0.063247* |
| **Emmetropic(Without TMDs)** | ***0.000131**** | *1.000000* | *0.063247* |  |

TA—the anterior part of the temporalis muscle; * significant difference.

# Table 15. Post hoc analysis for bioelectrical activity in the maximum voluntary clenching in intercuspal position (MM).

| **The Maximum Voluntary Clenching in Intercuspal Position MM** | **Myopia & TMDs** | **Myopia**  **(Without TMDs)** | **Emmetropic & TMDs** | **Emmetropic**  **(Without TMDs)** |
| --- | --- | --- | --- | --- |
| **Myopia & TMDs** |  | *0.120198* | ***0.000535**** | *0.178953* |
| **Myopia (Without TMDs)** | *0.120198* |  | *0.523634* | *1.000000* |
| **Emmetropic & TMDs** | ***0.000535**** | *0.523634* |  | *0.346324* |
| **Emmetropic(Without TMDs)** | *0.178953* | *1.000000* | *0.346324* |  |

MM—the superficial part of the masseter muscle;* significant difference

# Table 16. Post hoc analysis for bioelectrical activity in the maximum voluntary clenching in intercuspal position (SCM).

| **The Maximum Voluntary Clenching in Intercuspal Position SCM** | **Myopia & TMDs** | **Myopia**  **(Without TMDs)** | **Emmetropic & TMDs** | **Emmetropic**  **(Without TMDs)** |
| --- | --- | --- | --- | --- |
| **Myopia & TMDs** |  | ***0.000945**** | ***0.007631**** | *0.237028* |
| **Myopia (Without TMDs)** | ***0.000945**** |  | *1.000000* | *0.485553* |
| **Emmetropic & TMDs** | ***0.007631**** | *1.000000* |  | *1.000000* |
| **Emmetropic(Without TMDs)** | *0.237028* | *0.485553* | *1.000000* |  |

# SCM—the middle part of the sternocleidomastoid muscle; * significant difference.

# Table 17. Post hoc analysis for bioelectrical activity in the maximum voluntary clenching on dental cotton rolls in intercuspal position (SCM).

| **The Maximum Voluntary Clenching on Dental Cotton Rolls in Intercuspal Position**  **SCM** | **Myopia & TMDs** | **Myopia**  **(Without TMDs)** | **Emmetropic & TMDs** | **Emmetropic**  **(Without TMDs)** |
| --- | --- | --- | --- | --- |
| **Myopia & TMDs** |  | ***0.000108**** | ***0.001284**** | ***0.035931**** |
| **Myopia (Without TMDs)** | ***0.000108**** |  | *1.000000* | *0.692424* |
| **Emmetropic & TMDs** | ***0.001284**** | *1.000000* |  | *1.000000* |
| **Emmetropic(Without TMDs)** | ***0.035931**** | *0.692424* | *1.000000* |  |

SCM—the middle part of the sternocleidomastoid muscle; * significant difference.

# Table 18. Post hoc analysis for bioelectrical activity in the pain free maximum unassisted opening (DA).

| **The Pain Free Maximum Unassisted Opening**  **DA** | **Myopia & TMDs** | **Myopia**  **(Without TMDs)** | **Emmetropic & TMDs** | **Emmetropic**  **(Without TMDs)** |
| --- | --- | --- | --- | --- |
| **Myopia & TMDs** |  | *0.232540* | *1.000000* | *0.948410* |
| **Myopia (Without TMDs)** | *0.232540* |  | *1.000000* | ***0.002159**** |
| **Emmetropic & TMDs** | *1.000000* | *1.000000* |  | *0.186129* |
| **Emmetropic(Without TMDs)** | *0.948410* | ***0.002159**** | *0.186129* |  |

DA—the anterior belly of the digastric muscle; * significant difference.

# Table 19. Correlation results between refractive error and PSQI score, pressure pain threshold, bioelectrical activity scores between groups.

|  | | | **Myopia & TMDs** | | | **Myopia**  **(Without TMDs)** | | | **Emmetropic & TMDs** | | | **Emmetropic**  **(Without TMDs)** | | |
| --- | --- | --- | --- | --- | --- | --- | --- | --- | --- | --- | --- | --- | --- | --- |
|  | | | **R** | **t(N-2)** | **p** | **R** | **t(N-2)** | **p** | **R** | **t(N-2)** | **p** | **R** | **t(N-2)** | **p** |
| **Refractive Error (Dsph)** | **PSQI score** | | -0.12 | -0.75 | *0.45* | 0.01 | 0.04 | *0.97* |  |  |  |  |  |  |
|  | **Pressure**  **Pain**  **Threshold** | TA 1 | 0.15 | 0.98 | *0.33* | 0.13 | 0.77 | *0.44* |  |  |  |  |  |  |
|  |  | TA 2 | 0.02 | 0.15 | *0.88* | 0.21 | 1.27 | *0.21* |  |  |  |  |  |  |
|  |  | TA 3 | 0.02 | 0.10 | *0.92* | 0.12 | 0.74 | *0.46* |  |  |  |  |  |  |
|  |  | MM 1 | 0.04 | 0.28 | *0.78* | 0.10 | 0.59 | *0.56* |  |  |  |  |  |  |
|  |  | MM 2 | 0.21 | 1.36 | *0.18* | 0.11 | 0.69 | *0.50* |  |  |  |  |  |  |
|  |  | SCM | 0.18 | 1.18 | *0.25* | 0.09 | 0.55 | *0.58* |  |  |  |  |  |  |
|  |  | UT 1 | 0.16 | 1.00 | *0.32* | 0.11 | 0.67 | *0.51* |  |  |  |  |  |  |
|  |  | UT 2 | 0.07 | 0.44 | *0.66* | 0.18 | 1.12 | *0.27* |  |  |  |  |  |  |
|  |  | UT 3 | 0.06 | 0.38 | *0.70* | 0.17 | 1.01 | *0.32* |  |  |  |  |  |  |
|  |  | V1 | -0.05 | -0.29 | *0.78* | 0.19 | 1.15 | *0.26* |  |  |  |  |  |  |
|  |  | V2 | 0.08 | 0.49 | *0.62* | 0.19 | 1.17 | *0.25* |  |  |  |  |  |  |
|  |  | V3 | 0.18 | 1.14 | *0.26* | 0.15 | 0.90 | *0.37* |  |  |  |  |  |  |
|  | **Resting**  **Mandibular**  **Position** | TA | -0.16 | -1.02 | *0.31* | -0.26 | -1.66 | *0.11* |  |  |  |  |  |  |
|  |  | MM | -0.09 | -0.58 | *0.57* | -0.05 | -0.31 | *0.76* |  |  |  |  |  |  |
|  |  | SCM | -0.30 | -1.96 | *0.06* | -0.12 | -0.76 | *0.45* |  |  |  |  |  |  |
|  |  | DA | -0.20 | -1.18 | *0.25* | 0.09 | 0.55 | *0.59* |  |  |  |  |  |  |
|  | **The Maximum**  **Voluntary**  **Clenching**  **in Intercuspal Position** | TA | 0.11 | 0.69 | *0.49* | 0.08 | 0.52 | *0.60* |  |  |  |  |  |  |
|  |  | MM | 0.19 | 1.23 | *0.23* | -0.17 | -1.05 | *0.30* |  |  |  |  |  |  |
|  |  | SCM | **0.32** | **2.17** | ***0.04**** | **-0.31** | **-2.03** | ***0.04**** |  |  |  |  |  |  |
|  |  | DA | 0.25 | 1.45 | *0.16* | -0.27 | -1.75 | *0.09* |  |  |  |  |  |  |
|  | **The Maximum**  **Voluntary**  **Clenching**  **on Dental Cotton Rolls**  **in Intercuspal Position** | TA | 0.16 | 1.00 | *0.32* | 0.15 | 0.92 | *0.36* |  |  |  |  |  |  |
|  |  | MM | 0.24 | 1.58 | *0.12* | -0.17 | -1.04 | *0.30* |  |  |  |  |  |  |
|  |  | SCM | 0.25 | 1.61 | *0.11* | **-0.31** | **-2.03** | ***0.04**** |  |  |  |  |  |  |
|  |  | DA | 0.17 | 0.95 | *0.35* | -0.10 | -0.60 | *0.55* |  |  |  |  |  |  |
|  | **The Pain Free**  **Maximum**  **Unassisted Opening** | TA | 0.16 | 1.00 | *0.33* | -0.10 | -0.60 | *0.55* |  |  |  |  |  |  |
|  |  | MM | 0.09 | 0.59 | *0.56* | 0.00 | -0.01 | *0.99* |  |  |  |  |  |  |
|  |  | SCM | 0.04 | 0.25 | *0.81* | 0.00 | -0.03 | *0.98* |  |  |  |  |  |  |
|  |  | DA | -0.27 | -1.61 | *0.12* | -0.18 | -1.14 | *0.26* |  |  |  |  |  |  |

PSQI—the Pittsburgh Sleep Quality Index; TA—the temporalis muscle; MM—the masseter muscle; SCM—the sternocleidomastoid muscle; DA—the digastric muscle; UT—the upper trapezius; V1— the output of the ophthalmic nerve; V2— the output of the maxillary nerve; V3— the output of the mandibular nerve; Dsph—spherical diopter; R—the correlation coefficient; t(N-2)—the test value; * significant difference.

# Table 20. Correlation results between intraocular pressure and PSQI score, pressure pain threshold, bioelectrical activity scores between groups.

|  | | | **Myopia & TMDs** | | | **Myopia**  **(Without TMDs)** | | | **Emmetropic & TMDs** | | | **Emmetropic**  **(Without TMDs)** | | |
| --- | --- | --- | --- | --- | --- | --- | --- | --- | --- | --- | --- | --- | --- | --- |
|  | | | **R** | **t(N-2)** | **p** | **R** | **t(N-2)** | **p** | **R** | **t(N-2)** | **p** | **R** | **t(N-2)** | **p** |
| **Intraocular Pressure (mmHg)** | **PSQI score** | | 0.04 | 0.26 | *0.80* | 0.01 | 0.04 | *0.97* | 0.35 | 1.77 | *0.09* | **-0.38** | **-2.57** | ***0.01**** |
|  | **Pressure**  **Pain**  **Threshold** | TA 1 | 0.11 | 0.67 | *0.51* | -0.06 | -0.35 | *0.73* | 0.13 | 0.60 | *0.56* | 0.11 | 0.71 | *0.48* |
|  |  | TA 2 | 0.00 | -0.03 | *0.98* | 0.02 | 0.11 | *0.91* | -0.09 | -0.41 | *0.68* | 0.12 | 0.76 | *0.45* |
|  |  | TA 3 | 0.07 | 0.42 | *0.67* | -0.16 | -0.97 | *0.34* | 0.06 | 0.30 | *0.77* | 0.14 | 0.88 | *0.39* |
|  |  | MM 1 | 0.00 | -0.03 | *0.98* | 0.00 | 0.02 | *0.98* | 0.05 | 0.25 | *0.81* | 0.09 | 0.56 | *0.58* |
|  |  | MM 2 | 0.01 | 0.06 | *0.95* | 0.02 | 0.13 | *0.90* | 0.14 | 0.67 | *0.51* | 0.02 | 0.10 | *0.92* |
|  |  | SCM | 0.05 | 0.31 | *0.76* | 0.01 | 0.03 | *0.98* | -0.13 | -0.60 | *0.56* | 0.16 | 0.99 | *0.33* |
|  |  | UT 1 | 0.07 | 0.47 | *0.64* | 0.04 | 0.25 | *0.81* | -0.17 | -0.79 | *0.44* | 0.15 | 0.97 | *0.34* |
|  |  | UT 2 | -0.02 | -0.13 | *0.90* | 0.00 | 0.03 | *0.98* | -0.14 | -0.69 | *0.50* | 0.30 | 1.97 | *0.06* |
|  |  | UT 3 | 0.05 | 0.30 | *0.76* | -0.07 | -0.40 | *0.69* | -0.24 | -1.14 | *0.27* | 0.19 | 1.21 | *0.23* |
|  |  | V1 | 0.19 | 1.25 | *0.22* | 0.05 | 0.30 | *0.77* | -0.27 | -1.31 | *0.20* | -0.06 | -0.37 | *0.71* |
|  |  | V2 | 0.11 | 0.73 | *0.47* | -0.07 | -0.42 | *0.67* | -0.03 | -0.12 | *0.91* | -0.03 | -0.20 | *0.85* |
|  |  | V3 | -0.03 | -0.19 | *0.85* | -0.06 | -0.38 | *0.71* | -0.29 | -1.42 | *0.17* | -0.08 | -0.49 | *0.63* |
|  | **Resting**  **Mandibular**  **Position** | TA | 0.26 | 1.69 | *0.10* | -0.16 | -0.97 | *0.34* | 0.09 | 0.40 | *0.69* | 0.29 | 1.91 | *0.06* |
|  |  | MM | 0.10 | 0.65 | *0.52* | -0.15 | -0.93 | *0.36* | 0.04 | 0.21 | *0.84* | 0.28 | 1.84 | *0.07* |
|  |  | SCM | 0.11 | 0.70 | *0.49* | 0.19 | 1.17 | *0.25* | 0.04 | 0.20 | *0.84* | 0.10 | 0.65 | *0.52* |
|  |  | DA | -0.26 | -1.54 | *0.13* | 0.14 | 0.90 | *0.37* | 0.04 | 0.18 | *0.86* | **0.33** | **2.09** | ***0.04**** |
|  | **The Maximum**  **Voluntary**  **Clenching**  **in Intercuspal Position** | TA | 0.30 | 1.98 | *0.05* | 0.12 | 0.72 | *0.48* | 0.06 | 0.26 | *0.80* | -0.23 | -1.53 | *0.13* |
|  |  | MM | 0.20 | 1.29 | *0.21* | -0.18 | -1.10 | *0.28* | -0.06 | -0.28 | *0.78* | -0.27 | -1.75 | *0.09* |
|  |  | SCM | 0.18 | 1.17 | *0.25* | 0.04 | 0.25 | *0.80* | 0.06 | 0.30 | *0.77* | -0.16 | -1.03 | *0.31* |
|  |  | DA | 0.08 | 0.46 | *0.65* | 0.26 | 1.68 | *0.10* | 0.09 | 0.42 | *0.68* | -0.22 | -1.37 | *0.18* |
|  | **The Maximum**  **Voluntary**  **Clenching**  **on Dental Cotton Rolls**  **in Intercuspal Position** | TA | 0.27 | 1.76 | *0.09* | 0.04 | 0.26 | *0.79* | 0.07 | 0.33 | *0.74* | -0.28 | -1.84 | *0.07* |
|  |  | MM | 0.03 | 0.19 | *0.85* | -0.18 | -1.14 | *0.26* | 0.06 | 0.29 | *0.77* | -0.29 | -1.92 | *0.06* |
|  |  | SCM | -0.04 | -0.27 | *0.79* | 0.01 | 0.08 | *0.94* | -0.09 | -0.42 | *0.68* | -0.20 | -1.29 | *0.21* |
|  |  | DA | 0.01 | 0.06 | *0.95* | **0.33** | **2.14** | ***0.04**** | 0.21 | 1.03 | *0.31* | **-0.41** | **-2.73** | ***0.01**** |
|  | **The Pain Free**  **Maximum**  **Unassisted Opening** | TA | 0.13 | 0.81 | *0.43* | **0.33** | **2.14** | ***0.04**** | 0.08 | 0.38 | *0.71* | -0.04 | -0.23 | *0.82* |
|  |  | MM | 0.09 | 0.57 | *0.57* | 0.29 | 1.84 | *0.07* | 0.28 | 1.35 | *0.19* | -0.03 | -0.20 | *0.84* |
|  |  | SCM | -0.24 | -1.57 | *0.12* | 0.17 | 1.07 | *0.29* | 0.15 | 0.71 | *0.48* | -0.08 | -0.50 | *0.62* |
|  |  | DA | 0.09 | 0.53 | *0.60* | -0.01 | -0.05 | *0.96* | 0.22 | 1.06 | *0.30* | -0.23 | -1.38 | *0.18* |

PSQI—the Pittsburgh Sleep Quality Index; TA—the temporalis muscle; MM—the masseter muscle; SCM—the sternocleidomastoid muscle; DA—the digastric muscle; UT—the upper trapezius; V1— the output of the ophthalmic nerve; V2— the output of the maxillary nerve; V3— the output of the mandibular nerve; ; mmHg—conventional millimeters of mercury; R—the correlation coefficient; t(N-2)—the test value; * significant difference.

# Table 21. Correlation results between retinal thickness and PSQI score, pressure pain threshold, bioelectrical activity scores between groups.

|  | | | **Myopia & TMDs** | | | **Myopia**  **(Without TMDs)** | | | **Emmetropic & TMDs** | | | **Emmetropic**  **(Without TMDs)** | | |
| --- | --- | --- | --- | --- | --- | --- | --- | --- | --- | --- | --- | --- | --- | --- |
|  | | | **R** | **t(N-2)** | **p** | **R** | **t(N-2)** | **p** | **R** | **t(N-2)** | **p** | **R** | **t(N-2)** | **p** |
| **Retinal Thickness (μm)** | **PSQI score** | | **-0.51** | **-3.71** | ***0.00**** | -0.07 | -0.43 | *0.67* | 0.00 | -0.01 | *0.99* | 0.15 | 0.98 | *0.33* |
|  | **Pressure**  **Pain**  **Threshold** | TA 1 | 0.05 | 0.31 | *0.76* | -0.22 | -1.33 | *0.19* | -0.23 | -1.09 | *0.29* | 0.01 | 0.08 | *0.94* |
|  |  | TA 2 | 0.15 | 0.95 | *0.35* | -0.21 | -1.27 | *0.21* | 0.01 | 0.03 | *0.97* | -0.04 | -0.24 | *0.81* |
|  |  | TA 3 | 0.09 | 0.58 | *0.56* | -0.25 | -1.58 | *0.12* | -0.21 | -1.02 | *0.32* | -0.19 | -1.20 | *0.24* |
|  |  | MM 1 | 0.06 | 0.37 | *0.71* | -0.05 | -0.27 | *0.79* | 0.36 | 1.84 | *0.08* | -0.10 | -0.65 | *0.52* |
|  |  | MM 2 | 0.02 | 0.11 | *0.91* | 0.02 | 0.12 | *0.90* | 0.06 | 0.26 | *0.80* | 0.02 | 0.10 | *0.92* |
|  |  | SCM | -0.01 | -0.04 | *0.97* | -0.05 | -0.32 | *0.75* | 0.11 | 0.53 | *0.60* | -0.09 | -0.60 | *0.55* |
|  |  | UT 1 | -0.10 | -0.63 | *0.53* | 0.02 | 0.10 | *0.92* | 0.37 | 1.85 | *0.08* | 0.04 | 0.27 | *0.79* |
|  |  | UT 2 | -0.07 | -0.47 | *0.64* | -0.21 | -1.29 | *0.21* | **0.44** | **2.32** | ***0.03**** | 0.04 | 0.23 | *0.82* |
|  |  | UT 3 | -0.06 | -0.40 | *0.69* | -0.31 | -1.96 | *0.06* | 0.11 | 0.53 | *0.60* | -0.02 | -0.15 | *0.88* |
|  |  | V1 | -0.03 | -0.19 | *0.85* | -0.17 | -1.02 | *0.31* | 0.00 | 0.02 | *0.98* | -0.12 | -0.78 | *0.44* |
|  |  | V2 | 0.03 | 0.20 | *0.84* | -0.26 | -1.59 | *0.12* | -0.05 | -0.25 | *0.81* | -0.12 | -0.73 | *0.47* |
|  |  | V3 | 0.11 | 0.70 | *0.49* | 0.02 | 0.12 | *0.90* | 0.08 | 0.39 | *0.70* | -0.17 | -1.06 | *0.29* |
|  | **Resting**  **Mandibular**  **Position** | TA | -0.08 | -0.53 | *0.60* | 0.03 | 0.21 | *0.83* | **0.50** | **2.67** | ***0.01**** | -0.22 | -1.46 | *0.15* |
|  |  | MM | -0.25 | -1.60 | *0.12* | 0.20 | 1.27 | *0.21* | 0.11 | 0.50 | *0.62* | -0.09 | -0.60 | *0.55* |
|  |  | SCM | **-0.31** | **-2.07** | ***0.04**** | -0.20 | -1.24 | *0.22* | 0.06 | 0.29 | *0.78* | **0.46** | **3.30** | ***0.00**** |
|  |  | DA | -0.07 | -0.39 | *0.70* | -0.30 | -1.95 | *0.06* | 0.00 | 0.00 | *1.00* | **-0.46** | **-3.09** | ***0.00**** |
|  | **The Maximum**  **Voluntary**  **Clenching**  **in Intercuspal Position** | TA | -0.18 | -1.16 | *0.25* | -0.07 | -0.46 | *0.65* | -0.29 | -1.40 | *0.18* | 0.19 | 1.24 | *0.22* |
|  |  | MM | -0.29 | -1.89 | *0.07* | 0.26 | 1.64 | *0.11* | -0.24 | -1.15 | *0.26* | -0.24 | -1.58 | *0.12* |
|  |  | SCM | -0.11 | -0.68 | *0.50* | 0.14 | 0.86 | *0.40* | -0.15 | -0.70 | *0.49* | 0.03 | 0.21 | *0.84* |
|  |  | DA | 0.18 | 1.05 | *0.30* | 0.03 | 0.19 | *0.85* | -0.16 | -0.76 | *0.45* | -0.06 | -0.36 | *0.72* |
|  | **The Maximum**  **Voluntary**  **Clenching**  **on Dental Cotton Rolls**  **in Intercuspal Position** | TA | -0.18 | -1.19 | *0.24* | -0.08 | -0.52 | *0.61* | -0.27 | -1.31 | *0.20* | 0.14 | 0.89 | *0.38* |
|  |  | MM | -0.23 | -1.48 | *0.15* | 0.24 | 1.53 | *0.14* | -0.07 | -0.35 | *0.73* | -0.11 | -0.73 | *0.47* |
|  |  | SCM | -0.01 | -0.04 | *0.97* | 0.20 | 1.27 | *0.21* | -0.11 | -0.50 | *0.62* | 0.26 | 1.73 | *0.09* |
|  |  | DA | 0.21 | 1.21 | *0.24* | 0.10 | 0.63 | *0.53* | -0.06 | -0.30 | *0.77* | 0.21 | 1.28 | *0.21* |
|  | **The Pain Free**  **Maximum**  **Unassisted Opening** | TA | -0.27 | -1.78 | *0.08* | 0.10 | 0.63 | *0.53* | -0.04 | -0.20 | *0.84* | 0.18 | 1.19 | *0.24* |
|  |  | MM | 0.02 | 0.10 | *0.92* | 0.06 | 0.36 | *0.72* | 0.02 | 0.09 | *0.93* | -0.03 | -0.20 | *0.84* |
|  |  | SCM | 0.16 | 1.01 | *0.32* | 0.16 | 1.03 | *0.31* | -0.08 | -0.36 | *0.72* | -0.02 | -0.15 | *0.88* |
|  |  | DA | 0.19 | 1.07 | *0.29* | 0.27 | 1.74 | *0.09* | -0.19 | -0.91 | *0.37* | -0.13 | -0.79 | *0.44* |

PSQI—the Pittsburgh Sleep Quality Index; TA—the temporalis muscle; MM—the masseter muscle; SCM—the sternocleidomastoid muscle; DA—the digastric muscle; UT—the upper trapezius; V1— the output of the ophthalmic nerve; V2— the output of the maxillary nerve; V3— the output of the mandibular nerve; μm—micrometer; R—the correlation coefficient; t(N-2)—the test value; * significant difference.

# Table 22. Correlation results between choroidal thickness and PSQI score, pressure pain threshold, bioelectrical activity scores between groups.

|  | | | **Myopia & TMDs** | | | **Myopia**  **(Without TMDs)** | | | **Emmetropic & TMDs** | | | **Emmetropic**  **(Without TMDs)** | | |
| --- | --- | --- | --- | --- | --- | --- | --- | --- | --- | --- | --- | --- | --- | --- |
|  | | | **R** | **t(N-2)** | **p** | **R** | **t(N-2)** | **p** | **R** | **t(N-2)** | **p** | **R** | **t(N-2)** | **p** |
| **Choroidal Thickness (μm)** | **PSQI score** | | 0.01 | 0.08 | *0.94* | -0.06 | -0.37 | *0.71* | 0.36 | 1.79 | *0.09* | 0.20 | 1.28 | *0.21* |
|  | **Pressure**  **Pain**  **Threshold** | TA 1 | 0.18 | 1.18 | *0.25* | **0.56** | **4.05** | ***0.00**** | **-0.50** | **-2.74** | ***0.01**** | **0.49** | **3.54** | ***0.00**** |
|  |  | TA 2 | 0.03 | 0.20 | *0.84* | **0.57** | **4.17** | ***0.00**** | **-0.42** | **-2.17** | ***0.04**** | **0.41** | **2.84** | ***0.01**** |
|  |  | TA 3 | -0.09 | -0.55 | *0.59* | **0.49** | **3.34** | ***0.00**** | -0.32 | -1.58 | *0.13* | **0.45** | **3.19** | ***0.00**** |
|  |  | MM 1 | -0.06 | -0.38 | *0.71* | **0.59** | **4.33** | ***0.00**** | -0.23 | -1.09 | *0.29* | 0.15 | 0.97 | *0.34* |
|  |  | MM 2 | 0.06 | 0.40 | *0.69* | **0.43** | **2.88** | ***0.01**** | -0.25 | -1.20 | *0.24* | 0.19 | 1.24 | *0.22* |
|  |  | SCM | 0.02 | 0.14 | *0.89* | **0.48** | **3.29** | ***0.00**** | 0.06 | 0.27 | *0.79* | 0.28 | 1.81 | *0.08* |
|  |  | UT 1 | 0.00 | 0.00 | *1.00* | **0.54** | **3.88** | ***0.00**** | 0.25 | 1.21 | *0.24* | **0.46** | **3.29** | ***0.00**** |
|  |  | UT 2 | 0.12 | 0.76 | *0.45* | **0.43** | **2.89** | ***0.01**** | **0.48** | **2.55** | ***0.02**** | **0.37** | **2.50** | ***0.02**** |
|  |  | UT 3 | 0.01 | 0.09 | *0.93* | **0.44** | **2.95** | ***0.01**** | 0.20 | 0.98 | *0.34* | **0.40** | **2.74** | ***0.01**** |
|  |  | V1 | -0.13 | -0.83 | *0.41* | **0.61** | **4.58** | ***0.00**** | 0.18 | 0.84 | *0.41* | **0.36** | **2.45** | ***0.02**** |
|  |  | V2 | 0.19 | 1.19 | *0.24* | **0.53** | **3.74** | ***0.00**** | -0.01 | -0.04 | *0.97* | 0.28 | 1.83 | *0.08* |
|  |  | V3 | 0.08 | 0.49 | *0.62* | **0.63** | **4.84** | ***0.00**** | 0.09 | 0.41 | *0.68* | 0.22 | 1.45 | *0.15* |
|  | **Resting**  **Mandibular**  **Position** | TA | 0.10 | 0.65 | *0.52* | -0.05 | -0.32 | *0.75* | 0.25 | 1.21 | *0.24* | 0.13 | 0.81 | *0.42* |
|  |  | MM | -0.24 | -1.53 | *0.13* | 0.06 | 0.34 | *0.73* | -0.07 | -0.33 | *0.75* | 0.11 | 0.73 | *0.47* |
|  |  | SCM | -0.11 | -0.73 | *0.47* | 0.08 | 0.49 | *0.62* | 0.32 | 1.60 | *0.12* | 0.25 | 1.62 | *0.11* |
|  |  | DA | -0.14 | -0.84 | *0.41* | 0.21 | 1.32 | *0.20* | 0.13 | 0.62 | *0.54* | 0.24 | 1.46 | *0.15* |
|  | **The Maximum**  **Voluntary**  **Clenching**  **in Intercuspal Position** | TA | 0.04 | 0.22 | *0.83* | 0.21 | 1.35 | *0.19* | -0.20 | -0.96 | *0.35* | 0.27 | 1.76 | *0.09* |
|  |  | MM | -0.28 | -1.87 | *0.07* | -0.03 | -0.17 | *0.87* | -0.19 | -0.93 | *0.36* | 0.13 | 0.86 | *0.40* |
|  |  | SCM | 0.14 | 0.88 | *0.39* | -0.09 | -0.56 | *0.58* | -0.37 | -1.88 | *0.07* | 0.16 | 1.04 | *0.30* |
|  |  | DA | -0.07 | -0.39 | *0.70* | -0.15 | -0.95 | *0.35* | -0.17 | -0.82 | *0.42* | -0.10 | -0.62 | *0.54* |
|  | **The Maximum**  **Voluntary**  **Clenching**  **on Dental Cotton Rolls**  **in Intercuspal Position** | TA | -0.12 | -0.74 | *0.47* | 0.23 | 1.42 | *0.16* | -0.39 | -1.98 | *0.06* | 0.26 | 1.67 | *0.10* |
|  |  | MM | **-0.33** | **-2.23** | ***0.03**** | 0.07 | 0.45 | *0.66* | -0.18 | -0.84 | *0.41* | 0.19 | 1.22 | *0.23* |
|  |  | SCM | 0.08 | 0.50 | *0.62* | -0.03 | -0.15 | *0.88* | **-0.49** | **-2.61** | ***0.02**** | 0.18 | 1.13 | *0.27* |
|  |  | DA | -0.12 | -0.71 | *0.48* | 0.05 | 0.32 | *0.75* | -0.10 | -0.47 | *0.64* | -0.06 | -0.34 | *0.74* |
|  | **The Pain Free**  **Maximum**  **Unassisted Opening** | TA | 0.13 | 0.83 | *0.41* | 0.05 | 0.32 | *0.75* | -0.06 | -0.27 | *0.79* | -0.24 | -1.56 | *0.13* |
|  |  | MM | -0.05 | -0.34 | *0.73* | 0.20 | 1.25 | *0.22* | -0.21 | -1.03 | *0.32* | -0.21 | -1.37 | *0.18* |
|  |  | SCM | 0.22 | 1.41 | *0.17* | 0.28 | 1.77 | *0.09* | -0.19 | -0.89 | *0.38* | -0.16 | -1.00 | *0.32* |
|  |  | DA | **-0.41** | **-2.55** | ***0.02**** | **0.35** | **2.32** | ***0.03**** | 0.01 | 0.04 | *0.96* | **-0.50** | **-3.36** | ***0.00**** |

PSQI—the Pittsburgh Sleep Quality Index; TA—the temporalis muscle; MM—the masseter muscle; SCM—the sternocleidomastoid muscle; DA—the digastric muscle; UT—the upper trapezius; V1— the output of the ophthalmic nerve; V2— the output of the maxillary nerve; V3— the output of the mandibular nerve; μm—micrometer; R—the correlation coefficient; t(N-2)—the test value; * significant difference.

# Table 23. Correlation results between axial length and PSQI score, pressure pain threshold, bioelectrical activity scores between groups.

|  | | | **Myopia & TMDs** | | | **Myopia**  **(Without TMDs)** | | | **Emmetropic & TMDs** | | | **Emmetropic**  **(Without TMDs)** | | |
| --- | --- | --- | --- | --- | --- | --- | --- | --- | --- | --- | --- | --- | --- | --- |
|  | | | **R** | **t(N-2)** | **p** | **R** | **t(N-2)** | **p** | **R** | **t(N-2)** | **p** | **R** | **t(N-2)** | **p** |
| **Axial Length (mm)** | **PSQI score** | | -0.17 | -1.09 | *0.28* | **-0.32** | **-2.10** | ***0.04**** | 0.36 | 1.81 | *0.08* | -0.11 | -0.71 | *0.48* |
|  | **Pressure**  **Pain**  **Threshold** | TA 1 | -0.04 | -0.27 | *0.79* | -0.29 | -1.80 | *0.08* | -0.10 | -0.47 | *0.64* | **0.32** | **2.13** | ***0.04**** |
|  |  | TA 2 | 0.03 | 0.17 | *0.86* | -0.28 | -1.74 | *0.09* | -0.24 | -1.14 | *0.27* | 0.29 | 1.95 | *0.06* |
|  |  | TA 3 | 0.08 | 0.51 | *0.61* | **-0.35** | **-2.27** | ***0.03**** | -0.28 | -1.37 | *0.18* | 0.16 | 1.01 | *0.32* |
|  |  | MM 1 | 0.17 | 1.11 | *0.27* | -0.12 | -0.74 | *0.47* | -0.19 | -0.91 | *0.37* | 0.29 | 1.91 | *0.06* |
|  |  | MM 2 | 0.00 | 0.03 | *0.98* | -0.08 | -0.51 | *0.61* | -0.22 | -1.08 | *0.29* | **0.33** | **2.20** | ***0.03**** |
|  |  | SCM | -0.08 | -0.48 | *0.64* | -0.11 | -0.67 | *0.51* | -0.29 | -1.40 | *0.17* | 0.18 | 1.14 | *0.26* |
|  |  | UT 1 | 0.02 | 0.10 | *0.92* | 0.02 | 0.11 | *0.91* | -0.11 | -0.52 | *0.61* | 0.24 | 1.54 | *0.13* |
|  |  | UT 2 | 0.02 | 0.16 | *0.88* | -0.28 | -1.72 | *0.09* | -0.09 | -0.42 | *0.68* | 0.16 | 1.02 | *0.31* |
|  |  | UT 3 | 0.00 | -0.02 | *0.98* | **-0.34** | **-2.17** | ***0.04**** | -0.06 | -0.29 | *0.77* | 0.10 | 0.61 | *0.55* |
|  |  | V1 | 0.06 | 0.40 | *0.69* | -0.21 | -1.29 | *0.21* | -0.27 | -1.34 | *0.20* | 0.06 | 0.37 | *0.71* |
|  |  | V2 | 0.06 | 0.37 | *0.72* | -0.15 | -0.90 | *0.37* | -0.09 | -0.43 | *0.67* | 0.14 | 0.87 | *0.39* |
|  |  | V3 | -0.04 | -0.23 | *0.82* | -0.07 | -0.39 | *0.70* | -0.24 | -1.15 | *0.26* | 0.16 | 1.01 | *0.32* |
|  | **Resting**  **Mandibular**  **Position** | TA | -0.02 | -0.14 | *0.89* | -0.05 | -0.32 | *0.75* | -0.04 | -0.19 | *0.85* | -0.24 | -1.54 | *0.13* |
|  |  | MM | 0.11 | 0.70 | *0.49* | 0.02 | 0.10 | *0.92* | -0.30 | -1.46 | *0.16* | 0.10 | 0.66 | *0.51* |
|  |  | SCM | 0.11 | 0.68 | *0.50* | -0.16 | -1.03 | *0.31* | -0.33 | -1.65 | *0.11* | -0.17 | -1.10 | *0.28* |
|  |  | DA | 0.29 | 1.77 | *0.09* | **-0.37** | **-2.47** | ***0.02**** | **-0.47** | **-2.51** | ***0.02**** | -0.25 | -1.57 | *0.13* |
|  | **The Maximum**  **Voluntary**  **Clenching**  **in Intercuspal Position** | TA | -0.17 | -1.06 | *0.29* | 0.10 | 0.63 | *0.53* | -0.08 | -0.37 | *0.71* | 0.26 | 1.72 | *0.09* |
|  |  | MM | -0.08 | -0.50 | *0.62* | 0.31 | 2.00 | *0.05* | -0.10 | -0.46 | *0.65* | 0.04 | 0.26 | *0.80* |
|  |  | SCM | -0.12 | -0.75 | *0.46* | 0.12 | 0.76 | *0.45* | 0.08 | 0.36 | *0.73* | 0.08 | 0.53 | *0.60* |
|  |  | DA | -0.01 | -0.03 | *0.98* | **0.31** | **2.03** | ***0.04**** | -0.19 | -0.93 | *0.36* | -0.26 | -1.62 | *0.11* |
|  | **The Maximum**  **Voluntary**  **Clenching**  **on Dental Cotton Rolls**  **in Intercuspal Position** | TA | -0.20 | -1.30 | *0.20* | 0.09 | 0.55 | *0.59* | -0.02 | -0.10 | *0.92* | 0.09 | 0.56 | *0.58* |
|  |  | MM | -0.07 | -0.43 | *0.67* | **0.39** | **2.60** | ***0.01**** | 0.12 | 0.59 | *0.56* | 0.05 | 0.34 | *0.74* |
|  |  | SCM | 0.03 | 0.22 | *0.83* | 0.22 | 1.37 | *0.18* | 0.06 | 0.27 | *0.79* | 0.00 | 0.03 | *0.98* |
|  |  | DA | 0.08 | 0.43 | *0.67* | 0.10 | 0.64 | *0.52* | 0.19 | 0.89 | *0.38* | -0.12 | -0.71 | *0.48* |
|  | **The Pain Free**  **Maximum**  **Unassisted Opening** | TA | -0.20 | -1.29 | *0.21* | 0.10 | 0.64 | *0.52* | 0.05 | 0.22 | *0.83* | 0.12 | 0.77 | *0.45* |
|  |  | MM | 0.13 | 0.81 | *0.42* | 0.00 | 0.03 | *0.98* | -0.25 | -1.23 | *0.23* | **0.47** | **3.35** | ***0.00**** |
|  |  | SCM | 0.19 | 1.25 | *0.22* | -0.01 | -0.04 | *0.97* | -0.30 | -1.45 | *0.16* | **0.37** | **2.54** | ***0.01**** |
|  |  | DA | -0.07 | -0.41 | *0.68* | 0.14 | 0.85 | *0.40* | **-0.42** | **-2.14** | ***0.04**** | **0.63** | **4.76** | ***0.00**** |

PSQI—the Pittsburgh Sleep Quality Index; TA—the temporalis muscle; MM—the masseter muscle; SCM—the sternocleidomastoid muscle; DA—the digastric muscle; UT—the upper trapezius; V1— the output of the ophthalmic nerve; V2— the output of the maxillary nerve; V3— the output of the mandibular nerve; mm—millimeter; R—the correlation coefficient; t(N-2)—the test value; * significant difference.

# Table 24. Correlation results between PSQI score and pressure pain threshold, bioelectrical activity scores between groups.

|  | | | **Myopia & TMDs** | | | **Myopia**  **(Without TMDs)** | | | **Emmetropic & TMDs** | | | **Emmetropic**  **(Without TMDs)** | | |
| --- | --- | --- | --- | --- | --- | --- | --- | --- | --- | --- | --- | --- | --- | --- |
|  | | | **R** | **t(N-2)** | **p** | **R** | **t(N-2)** | **p** | **R** | **t(N-2)** | **p** | **R** | **t(N-2)** | **p** |
| **PSQI score** |  |  |  | | | | | | | | | | | |
|  | **Pressure**  **Pain**  **Threshold** | TA 1 | -0.13 | -0.85 | *0.40* | 0.02 | 0.12 | *0.90* | -0.06 | -0.34 | *0.74* | 0.00 | 0.02 | *0.98* |
|  |  | TA 2 | -0.15 | -0.93 | *0.36* | 0.12 | 0.70 | *0.49* | -0.19 | -1.06 | *0.30* | -0.14 | -0.90 | *0.37* |
|  |  | TA 3 | -0.13 | -0.85 | *0.40* | 0.13 | 0.76 | *0.45* | 0.00 | 0.00 | *1.00* | -0.14 | -0.92 | *0.36* |
|  |  | MM 1 | -0.18 | -1.14 | *0.26* | 0.00 | -0.02 | *0.98* | -0.08 | -0.44 | *0.66* | -0.18 | -1.16 | *0.25* |
|  |  | MM 2 | -0.21 | -1.33 | *0.19* | -0.12 | -0.72 | *0.48* | 0.04 | 0.24 | *0.81* | -0.06 | -0.37 | *0.71* |
|  |  | SCM | -0.16 | -1.02 | *0.31* | -0.09 | -0.55 | *0.58* | 0.17 | 0.94 | *0.35* | -0.05 | -0.34 | *0.74* |
|  |  | UT 1 | -0.17 | -1.10 | *0.28* | -0.09 | -0.52 | *0.61* | 0.22 | 1.26 | *0.22* | -0.04 | -0.23 | *0.82* |
|  |  | UT 2 | -0.14 | -0.88 | *0.39* | 0.06 | 0.34 | *0.73* | 0.27 | 1.52 | *0.14* | **-0.31** | **-2.09** | ***0.04**** |
|  |  | UT 3 | -0.14 | -0.86 | *0.39* | 0.11 | 0.66 | *0.52* | 0.17 | 0.93 | *0.36* | -0.09 | -0.58 | *0.57* |
|  |  | V1 | -0.05 | -0.34 | *0.74* | -0.03 | -0.15 | *0.88* | 0.24 | 1.29 | *0.21* | 0.07 | 0.47 | *0.64* |
|  |  | V2 | -0.07 | -0.42 | *0.67* | -0.13 | -0.82 | *0.42* | 0.27 | 1.50 | *0.15* | 0.05 | 0.31 | *0.76* |
|  |  | V3 | -0.08 | -0.50 | *0.62* | 0.01 | 0.08 | *0.94* | 0.07 | 0.37 | *0.71* | -0.03 | -0.19 | *0.85* |
|  | **Resting**  **Mandibular**  **Position** | TA | -0.03 | -0.18 | *0.86* | 0.08 | 0.51 | *0.62* | 0.07 | 0.40 | *0.69* | 0.04 | 0.25 | *0.81* |
|  |  | MM | 0.07 | 0.43 | *0.67* | 0.29 | 1.89 | *0.07* | -0.02 | -0.13 | *0.90* | -0.13 | -0.85 | *0.40* |
|  |  | SCM | 0.21 | 1.35 | *0.19* | 0.21 | 1.33 | *0.19* | 0.01 | 0.03 | *0.97* | 0.28 | 1.82 | *0.08* |
|  |  | DA | 0.24 | 1.47 | *0.15* | -0.13 | -0.84 | *0.41* | 0.03 | 0.18 | *0.86* | -0.31 | -1.99 | *0.05* |
|  | **The Maximum**  **Voluntary**  **Clenching**  **in Intercuspal Position** | TA | 0.14 | 0.90 | *0.37* | 0.03 | 0.17 | *0.86* | **-0.42** | **-2.54** | ***0.02**** | 0.29 | 1.91 | *0.06* |
|  |  | MM | 0.20 | 1.26 | *0.21* | 0.13 | 0.82 | *0.42* | **-0.52** | **-3.34** | ***0.00**** | **0.40** | **2.80** | ***0.01**** |
|  |  | SCM | 0.07 | 0.42 | *0.68* | 0.13 | 0.82 | *0.42* | **-0.45** | **-2.73** | ***0.01**** | -0.13 | -0.81 | *0.42* |
|  |  | DA | 0.04 | 0.25 | *0.80* | -0.08 | -0.47 | *0.64* | -0.29 | -1.63 | *0.11* | **0.34** | **2.15** | ***0.04**** |
|  | **The Maximum**  **Voluntary**  **Clenching**  **on Dental Cotton Rolls**  **in Intercuspal Position** | TA | 0.02 | 0.13 | *0.90* | -0.03 | -0.18 | *0.86* | -0.33 | -1.89 | *0.07* | **0.49** | **3.56** | ***0.00**** |
|  |  | MM | 0.09 | 0.58 | *0.56* | -0.07 | -0.41 | *0.68* | -0.26 | -1.49 | *0.15* | **0.50** | **3.66** | ***0.00**** |
|  |  | SCM | 0.00 | -0.01 | *0.99* | 0.06 | 0.40 | *0.69* | **-0.43** | **-2.64** | ***0.01**** | 0.05 | 0.34 | *0.74* |
|  |  | DA | 0.12 | 0.66 | *0.52* | -0.06 | -0.37 | *0.72* | -0.05 | -0.30 | *0.77* | **0.44** | **2.97** | ***0.01**** |
|  | **The Pain Free**  **Maximum**  **Unassisted Opening** | TA | 0.23 | 1.53 | *0.13* | -0.06 | -0.37 | *0.72* | 0.06 | 0.31 | *0.76* | -0.29 | -1.92 | *0.06* |
|  |  | MM | -0.08 | -0.52 | *0.61* | 0.19 | 1.19 | *0.24* | -0.17 | -0.93 | *0.36* | -0.18 | -1.18 | *0.24* |
|  |  | SCM | 0.08 | 0.48 | *0.64* | 0.23 | 1.43 | *0.16* | -0.34 | -2.01 | *0.05* | -0.15 | -0.96 | *0.34* |
|  |  | DA | 0.20 | 1.13 | *0.27* | 0.13 | 0.80 | *0.43* | -0.19 | -1.07 | *0.29* | **-0.39** | **-2.48** | ***0.02**** |

PSQI—the Pittsburgh Sleep Quality Index; TA—the temporalis muscle; MM—the masseter muscle; SCM—the sternocleidomastoid muscle; DA—the digastric muscle; UT—the upper trapezius; V1— the output of the ophthalmic nerve; V2— the output of the maxillary nerve; V3— the output of the mandibular nerve; R—the correlation coefficient; t(N-2)—the test value; * significant difference.

# Table 25. Correlation results between refractive error and mandibular range of motion.

|  |  |  | **Myopia & TMDs** | | | **Myopia**  **(Without TMDs)** | | | **Emmetropic & TMDs** | | | **Emmetropic**  **(Without TMDs)** | | |
| --- | --- | --- | --- | --- | --- | --- | --- | --- | --- | --- | --- | --- | --- | --- |
|  |  |  | **R** | **t(N-2)** | **p** | **R** | **t(N-2)** | **p** | **R** | **t(N-2)** | **p** | **R** | **t(N-2)** | **p** |
| **Refractive Error (Dsph)** | **Mandibular Range Of Motion (mm)** | **Pain Free Opening** | 0.00 | 0.02 | *0.99* | 0.04 | 0.22 | *0.82* |  |  |  |  |  |  |
|  |  | **Maximum Unassisted Opening** | -0.01 | -0.04 | *0.96* | 0.07 | 0.45 | *0.65* |  |  |  |  |  |  |
|  |  | **Maximum Assisted Opening** | 0.19 | 1.18 | *0.24* | 0.05 | 0.29 | *0.78* |  |  |  |  |  |  |
|  |  | **Mandibular Movement to The Right** | 0.20 | 1.27 | *0.21* | 0.19 | 1.21 | *0.24* |  |  |  |  |  |  |
|  |  | **Mandibular Movement to The Left** | 0.05 | 0.34 | *0.74* | 0.07 | 0.46 | *0.65* |  |  |  |  |  |  |
|  |  | **Protrusion** | -0.25 | -1.59 | *0.12* | 0.21 | 1.36 | *0.18* |  |  |  |  |  |  |

Dsph—spherical diopter; mm—millimeter; R—the correlation coefficient; t(N-2)—the test value; * significant difference.

# Table 26. Correlation results between intraocular pressure and mandibular range of motion.

|  |  |  | **Myopia & TMDs** | | | **Myopia**  **(Without TMDs)** | | | **Emmetropic & TMDs** | | | **Emmetropic**  **(Without TMDs)** | | |
| --- | --- | --- | --- | --- | --- | --- | --- | --- | --- | --- | --- | --- | --- | --- |
|  |  |  | **R** | **t(N-2)** | **p** | **R** | **t(N-2)** | **p** | **R** | **t(N-2)** | **p** | **R** | **t(N-2)** | **p** |
| **Intraocular Pressure (mmHg)** | **Mandibular Range Of Motion (mm)** | **Pain Free Opening** | 0.13 | 0.84 | *0.41* | -0.14 | -0.89 | *0.38* | -0.37 | -1.85 | *0.08* | 0.01 | 0.05 | *0.96* |
|  |  | **Maximum Unassisted Opening** | 0.18 | 1.11 | *0.27* | -0.12 | -0.73 | *0.47* | -0.28 | -1.36 | *0.19* | -0.03 | -0.15 | *0.88* |
|  |  | **Maximum Assisted Opening** | 0.18 | 1.11 | *0.27* | 0.00 | -0.01 | *0.99* | -0.24 | -1.17 | *0.25* | -0.05 | -0.30 | *0.77* |
|  |  | **Mandibular Movement to The Right** | 0.06 | 0.36 | *0.72* | 0.01 | 0.05 | *0.96* | 0.25 | 1.22 | *0.24* | **-0.45** | **-3.13** | ***0.00**** |
|  |  | **Mandibular Movement to The Left** | -0.03 | -0.20 | *0.84* | 0.13 | 0.81 | *0.42* | 0.02 | 0.08 | *0.93* | **-0.37** | **-2.44** | ***0.02**** |
|  |  | **Protrusion** | 0.04 | 0.25 | *0.80* | 0.13 | 0.82 | *0.42* | -0.20 | -0.98 | *0.34* | -0.28 | -1.78 | *0.08* |

mmHg—conventional millimeters of mercury; mm—millimeter; R—the correlation coefficient; t(N-2)—the test value; * significant difference.

# Table 27. Correlation results between retinal thickness an mandibular range of motion.

|  |  |  | **Myopia & TMDs** | | | **Myopia**  **(Without TMDs)** | | | **Emmetropic & TMDs** | | | **Emmetropic**  **(Without TMDs)** | | |
| --- | --- | --- | --- | --- | --- | --- | --- | --- | --- | --- | --- | --- | --- | --- |
|  |  |  | **R** | **t(N-2)** | **p** | **R** | **t(N-2)** | **p** | **R** | **t(N-2)** | **p** | **R** | **t(N-2)** | **p** |
| **Retinal Thickness (μm)** | **Mandibular Range Of Motion (mm)** | **Pain Free Opening** | 0.05 | 0.30 | *0.76* | -0.22 | -1.39 | *0.17* | 0.37 | 1.89 | *0.07* | 0.00 | 0.00 | *1.00* |
|  |  | **Maximum Unassisted Opening** | -0.07 | -0.46 | *0.65* | -0.21 | -1.30 | *0.20* | 0.14 | 0.68 | *0.50* | 0.06 | 0.38 | *0.71* |
|  |  | **Maximum Assisted Opening** | -0.16 | -1.01 | *0.32* | -0.19 | -1.17 | *0.25* | 0.15 | 0.71 | *0.49* | 0.05 | 0.28 | *0.78* |
|  |  | **Mandibular Movement to The Right** | 0.01 | 0.07 | *0.94* | -0.30 | -1.96 | *0.06* | **-0.45** | **-2.34** | ***0.03**** | 0.24 | 1.49 | *0.14* |
|  |  | **Mandibular Movement to The Left** | 0.10 | 0.64 | *0.52* | -0.01 | -0.04 | *0.97* | -0.26 | -1.27 | *0.22* | -0.13 | -0.78 | *0.44* |
|  |  | **Protrusion** | **0.40** | **2.66** | ***0.01**** | -0.12 | -0.73 | *0.47* | 0.22 | 1.08 | *0.29* | 0.11 | 0.71 | *0.48* |

μm—micrometer; mm—millimeter; R—the correlation coefficient; t(N-2)—the test value; * significant difference.

# Table 28. Correlation results between choroidal thickness and mandibular range of motion.

|  |  |  | **Myopia & TMDs** | | | **Myopia**  **(Without TMDs)** | | | **Emmetropic & TMDs** | | | **Emmetropic**  **(Without TMDs)** | | |
| --- | --- | --- | --- | --- | --- | --- | --- | --- | --- | --- | --- | --- | --- | --- |
|  |  |  | **R** | **t(N-2)** | **p** | **R** | **t(N-2)** | **p** | **R** | **t(N-2)** | **p** | **R** | **t(N-2)** | **p** |
| **Choroidal Thickness (μm)** | **Mandibular Range Of Motion (mm)** | **Pain Free Opening** | 0.03 | 0.20 | *0.84* | 0.13 | 0.78 | *0.44* | 0.19 | 0.90 | *0.38* | 0.29 | 1.83 | *0.07* |
|  |  | **Maximum Unassisted Opening** | -0.13 | -0.83 | *0.41* | 0.14 | 0.86 | *0.39* | 0.00 | -0.01 | *0.99* | 0.22 | 1.40 | *0.17* |
|  |  | **Maximum Assisted Opening** | -0.26 | -1.69 | *0.10* | 0.19 | 1.20 | *0.24* | -0.02 | -0.07 | *0.94* | 0.23 | 1.46 | *0.15* |
|  |  | **Mandibular Movement to The Right** | -0.27 | -1.70 | *0.10* | 0.06 | 0.37 | *0.72* | **-0.45** | **-2.34** | ***0.03**** | 0.17 | 1.06 | *0.30* |
|  |  | **Mandibular Movement to The Left** | -0.29 | -1.87 | *0.07* | -0.07 | -0.40 | *0.69* | **-0.52** | **-2.88** | ***0.01**** | 0.04 | 0.26 | *0.80* |
|  |  | **Protrusion** | **-0.36** | **-2.41** | ***0.02**** | 0.03 | 0.16 | *0.87* | -0.30 | -1.49 | *0.15* | 0.06 | 0.40 | *0.69* |

μm—micrometer; mm—millimeter; R—the correlation coefficient; t(N-2)—the test value; * significant difference.

# Table 29. Correlation results between axial length and mandibular range of motion.

|  |  |  | **Myopia & TMDs** | | | **Myopia**  **(Without TMDs)** | | | **Emmetropic & TMDs** | | | **Emmetropic**  **(Without TMDs)** | | |
| --- | --- | --- | --- | --- | --- | --- | --- | --- | --- | --- | --- | --- | --- | --- |
|  |  |  | **R** | **t(N-2)** | **p** | **R** | **t(N-2)** | **p** | **R** | **t(N-2)** | **p** | **R** | **t(N-2)** | **p** |
| **Axial Length (mm)** | **Mandibular Range Of Motion (mm)** | **Pain Free Opening** | 0.24 | 1.51 | *0.14* | -0.17 | -1.09 | *0.28* | -0.01 | -0.02 | *0.98* | 0.03 | 0.21 | *0.83* |
|  |  | **Maximum Unassisted Opening** | -0.03 | -0.19 | *0.85* | -0.17 | -1.05 | *0.30* | 0.02 | 0.10 | *0.92* | 0.09 | 0.55 | *0.59* |
|  |  | **Maximum Assisted Opening** | -0.08 | -0.48 | *0.64* | -0.14 | -0.87 | *0.39* | 0.13 | 0.59 | *0.56* | 0.08 | 0.48 | *0.63* |
|  |  | **Mandibular Movement to The Right** | 0.12 | 0.72 | *0.48* | **-0.39** | **-2.62** | ***0.01**** | 0.38 | 1.93 | *0.07* | 0.14 | 0.86 | *0.40* |
|  |  | **Mandibular Movement to The Left** | 0.07 | 0.46 | *0.65* | -0.13 | -0.83 | *0.41* | 0.02 | 0.09 | *0.93* | 0.00 | 0.02 | *0.99* |
|  |  | **Protrusion** | 0.24 | 1.53 | *0.13* | -0.30 | -1.94 | *0.06* | 0.22 | 1.08 | *0.29* | 0.14 | 0.86 | *0.40* |

mm—millimeter; R—the correlation coefficient; t(N-2)—the test value; * significant difference.

# Table 30. Correlation results between PSQI score and mandibular range of motion.

|  |  |  | **Myopia & TMDs** | | | **Myopia**  **(Without TMDs)** | | | **Emmetropic & TMDs** | | | **Emmetropic**  **(Without TMDs)** | | |
| --- | --- | --- | --- | --- | --- | --- | --- | --- | --- | --- | --- | --- | --- | --- |
|  |  |  | **R** | **t(N-2)** | **p** | **R** | **t(N-2)** | **p** | **R** | **t(N-2)** | **p** | **R** | **t(N-2)** | **p** |
| **PSQI score** | **Mandibular Range Of Motion (mm)** | **Pain Free Opening** | **-0.40** | **-2.67** | ***0.01**** | -0.04 | -0.27 | *0.79* | -0.16 | -0.88 | *0.38* | **0.50** | **3.52** | ***0.00**** |
|  |  | **Maximum Unassisted Opening** | -0.22 | -1.39 | *0.17* | -0.08 | -0.50 | *0.62* | **-0.38** | **-2.22** | ***0.03**** | **0.54** | **3.96** | ***0.00**** |
|  |  | **Maximum Assisted Opening** | -0.16 | -1.01 | *0.32* | -0.07 | -0.42 | *0.68* | -0.29 | -1.67 | *0.11* | **0.50** | **3.59** | ***0.00**** |
|  |  | **Mandibular Movement to The Right** | -0.13 | -0.83 | *0.41* | 0.17 | 1.08 | *0.29* | 0.02 | 0.11 | *0.91* | **0.50** | **3.55** | ***0.00**** |
|  |  | **Mandibular Movement to The Left** | -0.24 | -1.52 | *0.14* | 0.15 | 0.96 | *0.34* | 0.11 | 0.61 | *0.55* | **0.37** | **2.44** | ***0.02**** |
|  |  | **Protrusion** | -0.26 | -1.63 | *0.11* | 0.03 | 0.19 | *0.85* | -0.28 | -1.58 | *0.13* | **0.52** | **3.77** | ***0.00**** |

PSQI—the Pittsburgh Sleep Quality Index; mm—millimeter; R—the correlation coefficient; t(N-2)—the test value; * significant difference.
